# Dabigatran Combined With Benztropine Ameliorates Cobalt Chloride-Induced Parkinsonism in Rats, Restores Protease-Activated Receptor 1 (PAR1), and Mitigates Oxidative Stress

**DOI:** 10.7759/cureus.80486

**Published:** 2025-03-12

**Authors:** Sherine Abdelmissih, Laila Ahmed Rashed, Mohamed Sharif Ismail Negm, Walaa Mohamed Sayed, Hesham M Mahmoud, Soha Elmorsy

**Affiliations:** 1 Department of Medical Pharmacology, Faculty of Medicine Kasr Al-Ainy, Cairo University, Cairo, EGY; 2 Department of Medical Biochemistry and Molecular Biology, Faculty of Medicine Kasr Al-Ainy, Cairo University, Cairo, EGY; 3 Department of Pathology, Faculty of Medicine Kasr Al-Ainy, Cairo University, Cairo, EGY; 4 Department of Anatomy and Embryology, Faculty of Medicine Kasr Al-Ainy, Cairo University, Cairo, EGY

**Keywords:** adenosine, benztropine, cobalt chloride, dabigatran, dopamine, endothelin 1, glutathione, malondialdehyde, parkinsonism, protease-activated receptor 1

## Abstract

Background: The presumed implication of thromboembolic and oxidative stress pathways in parkinsonism guided the current research toward the exploration of the anticoagulant dabigatran etexilate (DE) as a thrombin inhibitor in the cobalt chloride (CoCl_2_)-induced parkinsonism (CIP) model, a model of significance to industrial toxins-related health issues.

Methods: Oral CoCl_2_ (12.5 mg/kg) was administered daily for 60 days, with the introduction of benztropine mesylate (BM) (10 mg/kg) and/or DE (3 mg/kg) on day 31. Rearing, postural instability, and pasta handling were evaluated, followed by histopathologic examination of the substantia nigra (SN) and striatum (STR). The expressions of brain dopamine receptor 2 (*D_2_*), adenosine* *receptor 1 (A_1_) and 2A (A_2A_), and protease-activated receptor 1 (PAR1), as well as the brain levels of dopamine (DA), endothelin 1 (ET1), malondialdehyde (MDA), and glutathione (GSH), were assessed.

Results: BM+DE restored the number of rears to the control level, compared to being reduced in the CIP model. BM+DE restored the first, second, third, and average displacement distances to the control level, compared to being reduced in the CIP model. BM+DE was superior to either BM or DE in restoring the time to finish eating pasta and the number of adjustments of forepaws while eating to control levels after being affected in the CIP model. BM+DE restored DA to the control level and was superior to DE in restoring D_2* *_to the control level. BM+DE was superior to BM in restoring A_1 _and A_2A_*,* increasing A_1_/A_2A _beyond the control level*. *BM+DE was superior to BM in restoring PAR1 and ET1 to control levels. BM+DE was superior to BM in restoring MDA to the control level and was superior to both BM and DE in increasing GSH beyond the control level. BM+DE exhibited the highest percentage of preserved neurons in SN, which was negatively correlated with MDA.

Conclusion: BM+DE offers a therapeutic potential for parkinsonism triggered by chronic exposure to CoCl_2_. The implication of thrombin-related factors and oxidative stress in the modulation of the dopaminergic-adenosinergic crosstalk is plausible.

## Introduction

Parkinson’s disease (PD) is the second most common neurodegenerative disorder after Alzheimer’s disease [1]. According to World Health Organization estimates, the elderly population will continue to increase, possibly to 17% of the population by 2050. Treatment for the management of neurodegenerative diseases will be concomitantly increased [2]. PD, formerly thought of as an age-related disorder, can also affect adults as young as 18 years old [3]. Early onset PD (age >40) is slowly progressive [4]. The incidence of PD is lower in females than in males [5].

Decreased dopaminergic signaling relative to higher cholinergic signaling triggers motor incoordination [6]. Parkinsonism is characterized by resting tremors, rigidity, bradykinesia, akathisia (inability to sit still), and an unstable posture. Parkinsonism (parkinsonian disorders) include PD, atypical parkinsonism, and secondary parkinsonism. Currently, the therapeutic approach to target parkinsonism is limited to reinforcing the dopaminergic pathway, for instance, by administering the dopamine (DA) precursor, levodopa. An alternative is to suppress the cholinergic pathway by administering central anticholinergic drugs. Anticholinergic antiparkinsonian drugs are used as monotherapy in the early stage of the disease, thereby delaying the need for levodopa [7]. One anticholinergic antiparkinsonian drug, benztropine, has a relatively favorable pharmacokinetic profile in addition to a long terminal half-life [8]. An efficient strategy to stop the progression of parkinsonism and arrest the death of dopaminergic neurons has not yet been found.

The adenosine receptor 1 (A1) is widely expressed in the basal ganglia, a dopaminergic-enriched brain area affected in parkinsonism, and there is an abundance of A2A expression in the brain. Both A1 and A2A are co-expressed in the striatum (STR), a part of the basal ganglia, and heterodimerized in ischemic and hypoxic brain conditions. Notably, A1-A2A heterodimers have distinct functions from those of either receptor alone, potentially creating separate pharmacologic targets [9,10]. Interestingly, A2A heterodimerization with the dopamine receptor 2 (D2) inhibits D2 [11]. Furthermore, A2A expressed on cholinergic neurons of the STR induces acetylcholine release, supporting the role of adenosinergic signaling in the dopaminergic-cholinergic imbalance in parkinsonism [12]. We aimed to explore the A1/A2A expression ratio, inspired by a previous exploration of pharmacologic agents targeting A1/A2A heterodimers in motor dysfunction [13]. Since A2A has constitutive activity even in the absence of adenosine, the A1/A2A expression ratio, but not the adenosine level, seemed relevant to our aim [14].

The implication of oxidative stress in parkinsonism is currently under extensive exploration. In parkinsonism, the oxidant/antioxidant imbalance in oxidative stress can be triggered by hypoxia and/or inflammation. In turn, oxidative stress can trigger inflammation and other brain pathologies. Oxidative stress in parkinsonism is characterized by increased lipid peroxides, such as malondialdehyde (MDA), especially in younger patients, but there is no relation to the duration of the disease [15]. The parkinsonism-associated increase in MDA is accompanied by reductions in several antioxidants, including glutathione (GSH) [16]. The depletion of GSH has been previously reported to correlate positively with disease severity [17].

Furthermore, along with oxidative stress, hypoxia triggers the release of the vasoactive amine endothelin 1 (ET1) [18]. In response to vasoactive compounds such as thrombin, ET1 mediates vasoconstriction, endothelial dysfunction, and platelet adhesion through the activation of protease-activated receptor 1 (PAR1). Apart from its known mitogenic activity, ET1 provokes and is provoked by proinflammatory cytokines, suggesting its possible involvement in neuroinflammatory disorders. As a component of oxidative stress, ET1 has also been shown to be elevated even in the early stages of parkinsonism [19].

Thrombin, a serine protease and well-known clotting factor, exerts its vascular responses by activating PAR1. The neuroinflammation triggered by thrombin is an integral part of parkinsonism [20]. The high expression of PAR1 in dopamine-enriched brain areas has prompted an investigation of the relationship between PAR1 and the dopaminergic system in parkinsonism [21]. The interaction between factors of the coagulation cascade and parkinsonism was hypothesized by Reuland and Church, and the tendency for thrombus formation may increase the likelihood of complications in parkinsonism [22]. The role of PAR1 in the progression of parkinsonism remains enigmatic. Dabigatran etexilate (DE), a direct thrombin inhibitor administered orally, reduces both free and clot-bound thrombin, which may be useful to elucidate the roles of PAR1 and ET1 in the pathogenesis of parkinsonism.

Environmental factors, including exposure to neurotoxic heavy metals, contribute to the increased risk of developing parkinsonism. Cobalt, released from cobalt chloride (CoCl_2_), is a neurotoxin that induces cell death by apoptosis and necrosis, peripheral sensory and motor defects, tremor, and cognitive decline [23]. The potential involvement of cobalt in parkinsonism is supported by its role in the formation of α-synuclein neurofibrils [24]. Cobalt is employed in the medical industry as a component in joint prostheses. Cobalt measured in the systemic circulation 10 years after hip prosthesis is 5- to 10-fold higher than the maximum permissible blood concentration [25]. Modeling parkinsonism using CoCl_2_ stems from its established induction of hypoxia-induced neurotoxicity both in vitro and in vivo, as well as its previous implication in neuronal damage, which was responsive to antioxidants [26,27]. CoCl_2_-induced parkinsonism (CIP) models also display oxidative stress, ET1 release, and inflammation [28-31]. Thus, CIP seemed appropriate to replicate many features of the pathogenic factors addressed in this study.

The present study aimed to explore the effects of long-term administration of DE alone or combined with BM in a male rat CIP model. Motor functions were assessed behaviorally, and dopaminergic signaling was examined along with the histopathology of the SN and STR. Adenosinergic signaling, thrombin-related factors, and oxidative stress were evaluated as well, being relevant to the presumed pathogenesis of parkinsonism as well as to DE dynamics. The novelty of our study stems not only from exploring the therapeutic benefit of DE in the CIP model, as compared to its combined use with BM but also from introducing a contemporary concept of integrating the abovementioned biomarkers to the established dopaminergic-adenosinergic crosstalk in the pathogenesis of parkinsonism.

## Materials and methods

Experimental animals and grouping

Adult male Wistar albino rats, 7-8 months old (200 - 250 g) were obtained from the Animal House of Faculty of Medicine, Kars Al-Ainy, Cairo University, Cairo, Egypt, and were kept, six rats per cage, in the acclimatization room at Medical Pharmacology Department, Faculty of Medicine Kasr Al-Ainy, Cairo University for 7 days before the start of the training sessions, under standard conditions (22±2°C room temperature; 40% - 55% relative humidity; 12-h light/dark cycle with lights on at 07:30 AM), and access to food and water ad libitum. Animal handling and experimentation were conducted according to the updated ARRIVE guidelines 2.0. All animal experiments were carried out in accordance with the Guide for the Care and Use of Laboratory Animals as adopted and promulgated by the U.S. National Institutes of Health. All experimental procedures were reviewed and approved by the Institutional Animal Care and Use Committee, Cairo University (CU-III-F-81-22).

Adult male Wistar albino rats (N=54) were equally divided into nine groups of six rats each. All control rats (groups I-V) were administered 12.5 mL/kg distilled water (DW) (vehicle control for CoCl_2_) orally each day for 60 days. In group I, from days 31-60, 10 mL/kg DW was injected intraperitoneally (IP) (control for benztropine mesylate (BM)). Group II additionally received 15 mL/kg dimethyl sulfoxide (DMSO; Amazon, USA) orally from days 31-60 (control for DE). Group III received 10 mg/kg BM (Flagship Biotech International Private Limited, India) administered intraperitoneally (IP) from days 31-60 [32]. The IP route of administration for BM was chosen to avoid the slowing effect of multiple doses on the gastric emptying rate in rats, in an attempt to reduce alterations of oral absorption of CoCl_2_ and/or DE when combined with BM in the parkinsonism model groups [33]. DE-treated rats (group IV) were additionally administered daily oral DE (3 mg/kg) (Raxuter Chemicals, India) on days 31-60, followed by 10 mL/kg DW injected IP as in group I [34]. From days 31-60, BM+DE-treated rats (group V) were administered 3 mg/kg DE orally as in group IV, and 10 mg/kg BM injected immediately after DE administration, as in group III (Table 1).

**Table 1:** Experimental design and animal grouping. DW: distilled water; DMSO: dimethyl sulfoxide; BM: benztropine mesylate, DE: dabigatran etexilate, CoCl_2_: cobalt chloride, IP: intraperitoneal

Group (n = 6)	Agent(s)		Dose	Timing	Route of administration
Control	12.5 mL/kg DW (60 days)	DW (30 days)	10 mL/kg		IP
DMSO		DMSO (30 days)	15 mL/kg		Oral
BM-treated		BM (30 days)	10 mg/kg	1 hour after DW	IP
DE-treated		DE (30 days)	3 mg/kg	1 hour after DW	Oral
		DW (30 days)	10 mL/kg	Immediately after DE	IP
BM+DE-treated		BM (30 days)	10 mg/kg	Immediately after DE	IP
		DE (30 days)	3 mg/kg	1 hour after DW	Oral
CoCl_2_-induced parkinsonism	CoCl_2_ (60 days)	CoCl_2_ (30 days)	12.5 mg/kg		Oral
		DW (30 days)	10 mL/kg	1 hour after CoCl_2_	IP
BM-treated model		BM (30 days)	10 mg/kg	1 hour after CoCl_2_	IP
DE-treated model		DE (30 days)	3 mg/kg	1 hour after CoCl_2_	Oral
		DW (30 days)	10 mL/kg	Immediately after DE	IP
BM+DE-treated model		BM (30 days)	10 mg/kg	Immediately after DE	IP
		DE (30 days)	3 mg/kg	1 hour after CoCl_2_	Oral

CoCl_2_ (Sigma-Aldrich, USA) was used to induce parkinsonism (CIP). Groups VI-IX were orally administered CoCl_2_ (12.5 mg/kg) for 60 days [35]. In group VI, from days 31-60, rats were injected IP with DW 1 h after receiving CoCl_2_ (as in group I). From days 31-60, BM-treated CIP model rats (group VII) received BM-injected IP 1 h after receiving CoCl_2_ (as in group III). The DE-treated CIP model rats (group VIII) received DE orally from days 31-60, 1 h after CoCl_2_, followed by IP DW as in group IV. BM+DE-treated model rats (group IX) were administered CoCl_2_ and received BM+DE from days 31-60, as in group V (Table 1). Both oral and IP routes were adopted so the pattern of administration was consistent in all studied groups. All treatments were given after an overnight fast.

Assessments of motor function

On day 60 after the treatments, the following three tests were performed to assess motor function.

Rearing Behavior

Rats were placed in a clear cylinder (30 cm in height and 15 cm in diameter) for 5 min. In this assay, the rat will try to contact the cylinder wall by standing on its hind paws (rearing) as part of its exploratory behavior. A rearing movement is defined when both forepaws are on the floor of the cylinder, elevated to the wall, and then return to the floor. The assay was video-recorded, and the number and maximum duration of rears were calculated. The cage was cleaned with 5% ethanol to remove olfactory cues in between individual rats [36,37].

Postural Instability Test (PIT)

The PIT was performed according to the method described by Woodlee et al. [38]. This test is initiated only after the rat is comfortable with relaxed muscles while being held. Each rat is held vertically by the hindlimbs so that its forelimbs can touch a piece of sandpaper kept on the table to prevent slipping or dragging. One forelimb is restrained lightly against the rat’s torso, and its nose is aligned at the zero line. The rat moves forward gradually, advancing the center of gravity. The animal initiates a catch-up step to restore its balance, and the new position of the rat’s nose is recorded. After stepping twice, the average of the two distances from the initial zero line and the new line attained by the rat’s nose is calculated and considered as the first displacement distance (cm) required to regain its balance. This procedure was repeated thrice for each forelimb to yield three displacement distances (cm), by bringing the animal back to the zero position after each round. First, second, and third displacement distances for the right and left forepaws were compared, as well as the average displacement distances for the right and left forepaws. The percent change from first to third displacement for each of the right and left forepaws was also calculated.

Pasta Handling Test

Pasta (spaghetti) was incorporated in rat feed for 2 weeks prior to testing; six pieces of pasta, 4 cm long and 1.5 mm wide, were provided to each rat. On the test day, after an overnight fast, each tested rat was isolated from its cage mates and put in a plexiglass cage. They were provided three pieces of the same type of pasta. A video recording was initiated with the lens of the camera positioned lower than the level of the cage floor tilted slightly upward to facilitate visualization of postural instability. During recording, one piece of pasta was presented to each rat per session, for three sessions. It was dropped in the front area of the cage so that the rat would face the video camera. Pasta strands were marked at 1.5 cm segments to facilitate visualization of strand movement. If the rat moved away from the scope of the camera lens, the trial was ignored. The time to pick up the pasta stick (TPP), the time to finish eating it (TTF) and the number of times each forepaw was adjusted per piece (NTAF) were obtained from the video recording. Recording was paused once the rat stopped and was resumed once it started eating again. Forepaw movements were classified at the onset of eating as either a grasp or a guide. The grasp forepaw was held at the lower part of the pasta piece in a whole-paw grasp, while the guide forepaw was used more often, with the pasta segment held near the mouth in between one or more digits and the thumb nub. The adjustment was defined as any visible release-regrasp of the pasta piece or reformation of paw-hold of the pasta piece by extension-flexion and/or abduction-adduction of the digits [39,40]. The maximum duration of recording was 15 min.

Quantitative reverse transcription-polymerase chain reaction (qRT-PCR) of D2, A1, A2A, and PAR1 receptors

On day 60, and after assessments of motor function, rats were euthanized by decapitation under general anesthesia (mixture of ketamine (70 mg/kg) and xylazine (7 mg/kg) delivered intravenously). Their brains were removed from the skull, and the two hemispheres of each rat were separated and randomly assigned for biochemical and histopathologic assessments. To illustrate the dopamine-adenosine crosstalk and the involvement of PAR1 receptors in the potential therapeutic role of DE, alone or combined with BM, as well as in the CIP model, we used quantitative real-time PCR analysis to determine the gene expression of D2, A1, A2A, and PAR1 receptors. Rat brain hemispheres were washed in phosphate-buffered saline using vigorous titration, collected by centrifugation, and stored at −22°C. Total RNA was isolated using the ToTALLY RNA kit (Ambion, Thermo Fisher Scientific, USA) according to the manufacturer’s instructions. The RNA pellet was extracted with ethanol, dried, and dissolved in 10 μl of water. RNA concentration was determined by absorbance at 260 nm. Total RNA (100 ng) was reverse-transcribed (50°C for 30 min) and amplified using the Titan RT-PCR (Boehringer Ingelheim, Germany) system. Single-stranded cDNA products were denatured and subjected to PCR amplification (30 cycles). Each PCR cycle consisted of denaturing at 94°C for 45 s, annealing at 64°C for 45 s, and extension at 72°C for 60 s. The amplified products were separated by 1.2% agarose gel electrophoresis, and the bands were visualized using ethidium bromide. The RNA sequences were analyzed in triplicate using a Perkin Elmer/Applied Biosystems Division 7700 Sequence Detector (USA). The primers were designed using the Perkin Elmer/Applied Biosciences Primer Express software (USA) based on mRNA sequences reported in the National Center for Biotechnology Information database (USA) (Table 2).

**Table 2:** Gene-specific primer pairs for quantitative real-time polymerase chain reaction of dopamine receptor type 2 (D2), adenosine receptor type 1 (A1), adenosine receptor type 2A (A2A), and protease-activated receptor 1 (PAR1). Beta-actin was used as the control housekeeping gene. D2: dopamine receptor 2; A1: adenosine receptor 1, A2A: adenosine receptor 2, PAR1: protease-activated receptor 1

Gene	Forward primer sequence	Reverse primer sequence
D_2_	5'CTT CAC ATG GCT GGG CTA TGT3'	5'GCG GAA CTC GAT GTT GAA GG3'
A_1_	5' CTC CAT TCT GGC TCT GCT CG 3'	5'ACA CTG CCG TTG GCT CTC C3'
A_2A_	5'CCA TGC TGG GCT GGA ACA3'	5'GAA GGG GCA GTA ACA CGA ACG3'
PAR1	5'ACTATTTCTCCGCCTTCTCCGCCAT3'	5'TCACGCAGACGCAGAGGAGGAGGTAAGC3'
Beta-actin	5'GGTCGGTGTGAACGGATTTGG3'	5'ATGTAGGCCATGAGGTCCACC3'

ELISA determination of brain levels of DA, ET1, MDA, and GSH

To explore whether DE, alone or combined with BM, as well as CoCl_2_ can affect brain DA levels by altering inflammatory and oxidative stress responses, brain levels of DA (ng/50 mg brain tissue), ET1 (pg/50 mg brain tissue), MDA (pmol/50 mg brain tissue), and GSH (mmol/50 mg brain tissue were determined according to the manufacturers’ protocols. DA and ET1 ELISA kits were purchased from Cusabio (USA). MDA and GSH ELISA kits were purchased from MyBioSource (USA).

Preparation of paraffin sections and hematoxylin and eosin (H&E) staining of striatum and substantia nigra

To identify parkinsonism pathognomonic neuronal lesions in CIP model rats and determine whether they were targeted using DE, alone or combined with BM, brain hemispheres were sectioned coronally. The caudal and ventral halves contained the SN and STR, respectively, in which differential damage to either dopaminergic cell bodies or terminals can occur [41]. Brain sections were fixed in 10% buffered formalin and embedded in paraffin. Multiple coronal sections (5 μm) of the SN and STR were stained using H&E and examined to visualize dopaminergic cell bodies and dopaminergic terminals, respectively. Visualization of H&E-stained sections was performed by two investigators who were blinded to the animal grouping. A third external examiner concurred and ascertained the microscopic findings.

Histomorphometric analysis of substantia nigra

To determine the extent of dopaminergic neuronal loss and recovery, the percentage of preserved neurons in the SN was calculated in 10 non-overlapping microscopic fields. The count of intact neurons in H&E-stained slices of the SN was quantified using the Leica Qwin 500 (Cambridge, UK) image analysis system and a computer that adjusted the camera sensitivity (at magnification ×400). The percentage of preserved neurons was measured in relation to the global count of neuronal cells. Each slide was examined by two investigators who were blinded to the animal grouping. A third external examiner concurred and ascertained the neuron count.

Statistical analysis

The sample size was calculated using GPower v.3.1.9.4, adopting an effect size (d) of 2.2, α of 0.05, and power of 0.95. The experimental data were analyzed using GraphPad Prism software v. 10.1.2 (GraphPad Software, Inc., San Diego, CA, USA). Data are presented as mean ± standard deviation (SD) or median and interquartile range (IQR). Comparisons between groups were carried out using the Kruskal-Wallis test, followed by Dunn’s post hoc test for non-normally distributed data, and analysis of variance (ANOVA) followed by Tukey’s post hoc test for normally distributed data. Correlations were performed using Spearman’s rho coefficient. Differences were considered statistically significant when P < 0.05.

## Results

As the results of the DMSO group (II) did not differ significantly from control rats receiving DW (I), the comparisons in the text refer to the control group receiving DW (I).

DE, alone or combined with BM, restored the number of rears to control level

**Figure 1:**
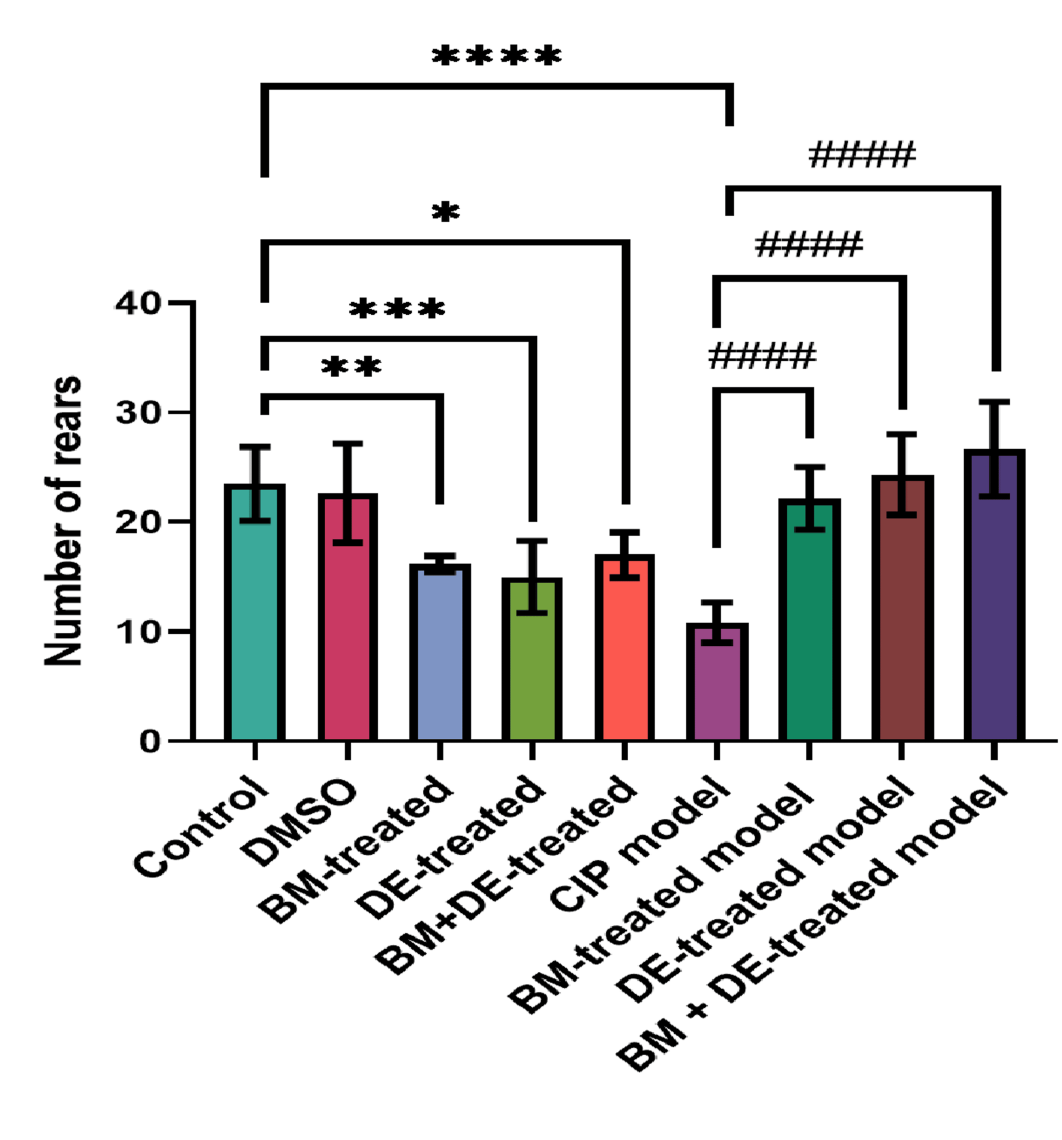
BM+DE improved number of rears (n=6). Data are expressed as mean ± SD. DMSO: dimethyl sulfoxide; CIP: cobalt chloride-induced parkinsonism; BM: benztropine mesylate; DE: dabigatran etexilate *Ρ < 0.05, **Ρ < 0.01, ***Ρ < 0.001, ****Ρ < 0.0001 vs controls; # # # #Ρ < 0.0001 vs CIP.

CoCl_2_ impaired rearing behavior in rats as detected by a significantly reduced number of rears relative to controls ((10.83±1.84 vs. 23.5±3.39) (F (8,45) = 16.26, ρ < 0.0001)) (Figure 1) with no significant changes of the maximum rearing duration. The BM-, DE-, and BM+DE-treated model groups exhibited significant improvements in rearing behavior, as evidenced by increased number of rears compared to the CIP model ((22.17±2.86, 24.33±3.67, and 26.67±4.32, respectively) (F (8,45) = 16.26, Ρ < 0.0001)), to be restored to control levels in the three treated CIP model groups (Figure 1). No significant differences were detected comparing BM-, DE-, and BM+DE-treated model groups. Surprisingly, the administration of BM-, DE-, or their combination to controls reduced the number of rears ((16.17±0.75, 15.00±3.29, and 17.00±2.10, respectively) (F (8,45) = 16.26, Ρ < 0.0001)) (Figure 1).

DE, alone or combined with BM, restored the displacement distances of both forepaws to control levels

**Figure 2:**
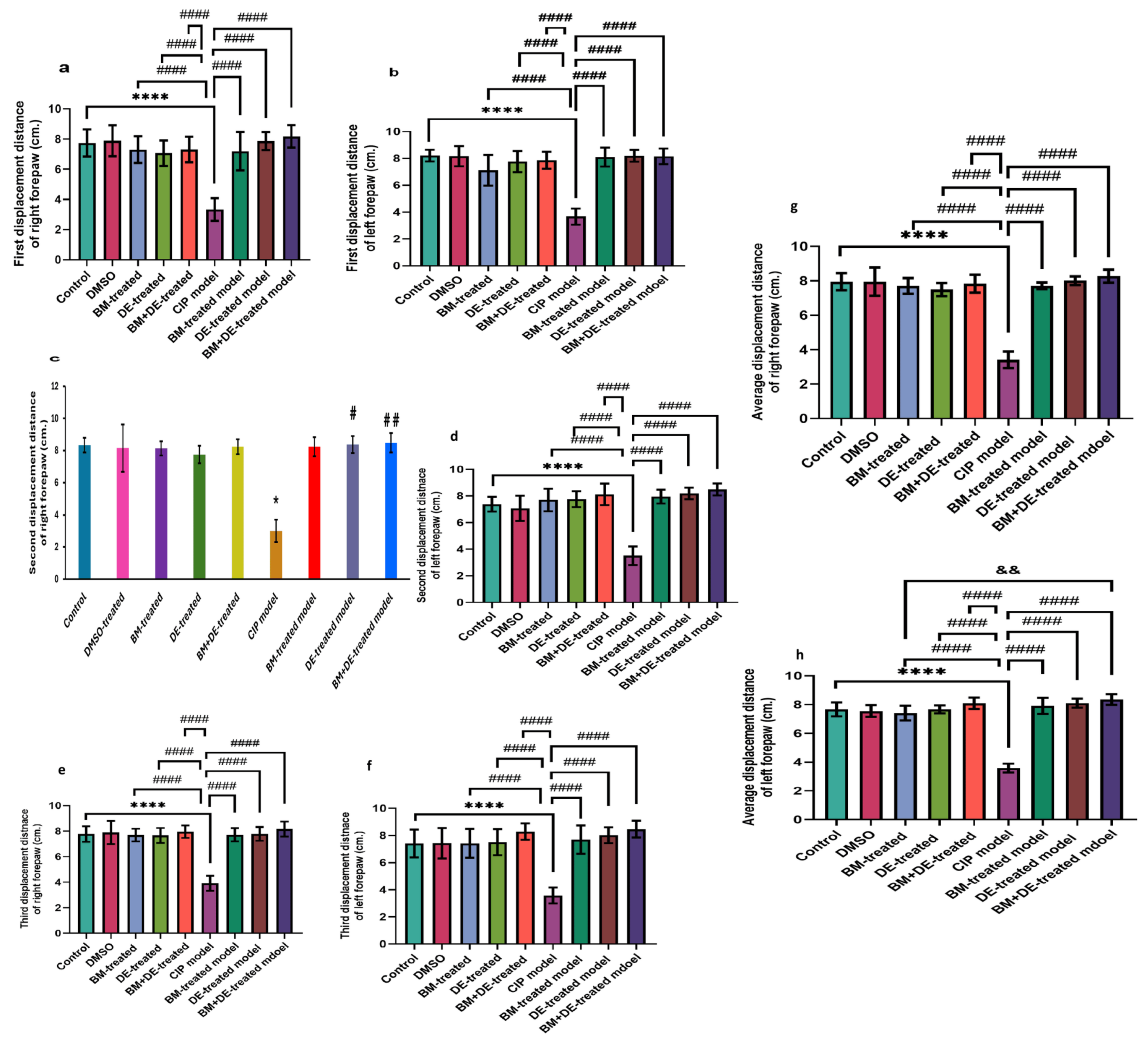
BM+DE improved postural instability (n=6). a) First displacement distance of the right forepaw; b) First displacement distance of the left forepaw; c) Second displacement distance of the right forepaw; d) Second displacement distance of the left forepaw; e) Third displacement distance of the right forepaw; f) Third displacement distance of the left forepaw; g) Average displacement distance of the right forepaw; h) Average displacement distance of the left forepaw. Data are expressed as mean ± SD. DMSO: dimethyl sulfoxide; CIP: cobalt chloride-induced parkinsonism; BM: benztropine mesylate; DE: dabigatran etexilate. * Ρ < 0.05, ****Ρ < 0.0001 vs controls; #Ρ < 0.05, # #Ρ < 0.01, # # # #Ρ < 0.0001 vs CIP; & &Ρ < 0.01 vs BM+DE-treated model.

CoCl_2_ significantly reduced the first displacement distances of both the right and the left forepaws ((3.33±0.75 and 3.67±0.61 cm, respectively, vs. control 7.74±0.90 and 8.22±0.44 cm, respectively) (F (8,45) = 16.09, Ρ < 0.0001; F (8,45) = 26.08, Ρ < 0.0001, respectively)) (Figures 2a-2b). Similarly, CoCl_2_ significantly reduced the second displacement distances of both the right ((3±0.71 vs. 8.34±0.45 cm) (t (9) = 21.20, Ρ < 0.05)) (Figure 2c) and the left forepaws ((3.51±0.69 vs. 7.39±0.56 cm) (F (8,45) = 30.12, Ρ < 0.0001)) (Figure 2d). This was also the case comparing the third displacement distance of the right and the left forepaws ((3.92±0.59 and 3.58±0.59 cm, respectively, vs. control 7.77±0.61 and 7.41±1.03 cm, respectively) (F (8,45) = 28.57, Ρ < 0.0001 and F (8,45) = 16.63, Ρ < 0.0001, respectively)) (Figures 2e-2f). Averaging the three displacement distances of each forepaw emphasized the significant impairments induced by CoCl_2_, as they were significantly reduced relative to controls ((3.42±0.48 and 3.59±0.31 cm, respectively, vs. 7.95±0.50 and 7.67±0.49 cm, respectively) (F (8,45) = 60.29, Ρ < 0.0001 and F (8,45) = 74.23, Ρ < 0.0001, respectively)) (Figures 2g-2h). Relative to the untreated model, BM, DE, and their combination significantly increased the first displacement distance of the right forepaw ((7.20±1.27, 7.87±0.59, and 8.18±0.74 cm, respectively) (F (8,45) = 16.09, Ρ < 0.0001)) (Figure 2a). Similarly, the first displacement distance of the left forepaw was significantly increased in each of the BM-, DE, and BM+DE-treated model groups ((2.21±0.71, 8.20±0.44, and 8.16±0.58 cm, respectively) (F (8,45) = 26.08, Ρ < 0.0001)) (Figure 2b). The first displacement distances of both forepaws were restored to control levels in all treated model groups. No significant differences were detected among the BM-, DE-, and BM+DE-treated model groups. Both DE and BM+DE, but not BM, increased the second displacement distances of the right forepaw relative to the untreated CIP model ((8.37±0.53 and 8.49±0.61 cm, respectively) (t (9) = 21.20, ρ < 0.05) and t (9) = 21.20, Ρ < 0.01, respectively)), restoring them to control levels. In this context, the DE-treated model group did not significantly differ from the BM+DE-treated model group (Figure 2c). Notably, the second displacement distance of the right forepaw in the BM-treated model did not significantly differ from that of controls. The second displacement distances of the left forepaw were significantly increased after BM, DE, and BM+DE treatments of the CIP model ((7.95±0.52, 8.19±0.44, and 8.49±0.45 cm, respectively) (F (8,45) = 30.12, Ρ < 0.0001)), restoring them to control levels. No significant variations were found comparing BM-, DE-, and BM+DE-treated model groups (Figure 2d). BM, DE, and their combination substantially improved the third displacement distance of the right forepaw, showing significantly higher values than the untreated CIP model ((7.72±0.52, 7.79±0.53 and 8.16±0.59, respectively) (F (8,45) = 28.57, Ρ < 0.0001)), restoring them to control levels (Figure 2e). Such improvement also applied to the third displacement distance of the left forepaw; BM, DE, and their combination increased the distance significantly relative to the untreated CIP model ((7.70±1.05, 8.02±0.58, and 8.47±0.61 cm, respectively) (F (8,45) = 16.63, Ρ < 0.0001)), restoring them to control levels (Figure 2f). Notably, the enhanced third displacement distances of both forepaws did not vary significantly among the three treated model groups. Treatment with BM, DE, or their combination significantly increased the average displacement distance of the right forepaw ((7.72±0.19, 8.01±0.25, and 8.28±0.38 cm, respectively) (F (8,45) = 60.29, Ρ < 0.0001)). Such improvements resulted in their restoration to control levels (Figure 2g) with no significant variations among the three treated model groups. Similarly, the significant improvements in the average displacement distance of the left forepaw exerted by BM, DE, and their combination were remarkable ((7.92±0.56, 8.11±0.31, and 8.35±0.37 cm, respectively) (F (8,45) = 74.23, Ρ < 0.0001)). Such improvements resulted in their restoration to control levels (Figure 2h). In this context, no significant differences were detected among the three treated model groups. The percent change from the first to the third displacement distances of the right and left forepaw did not show any significant differences among all studied groups.

DE combined with BM restored TTF to the control level and improved NTAF beyond the control level in the pasta handling test

**Figure 3:**
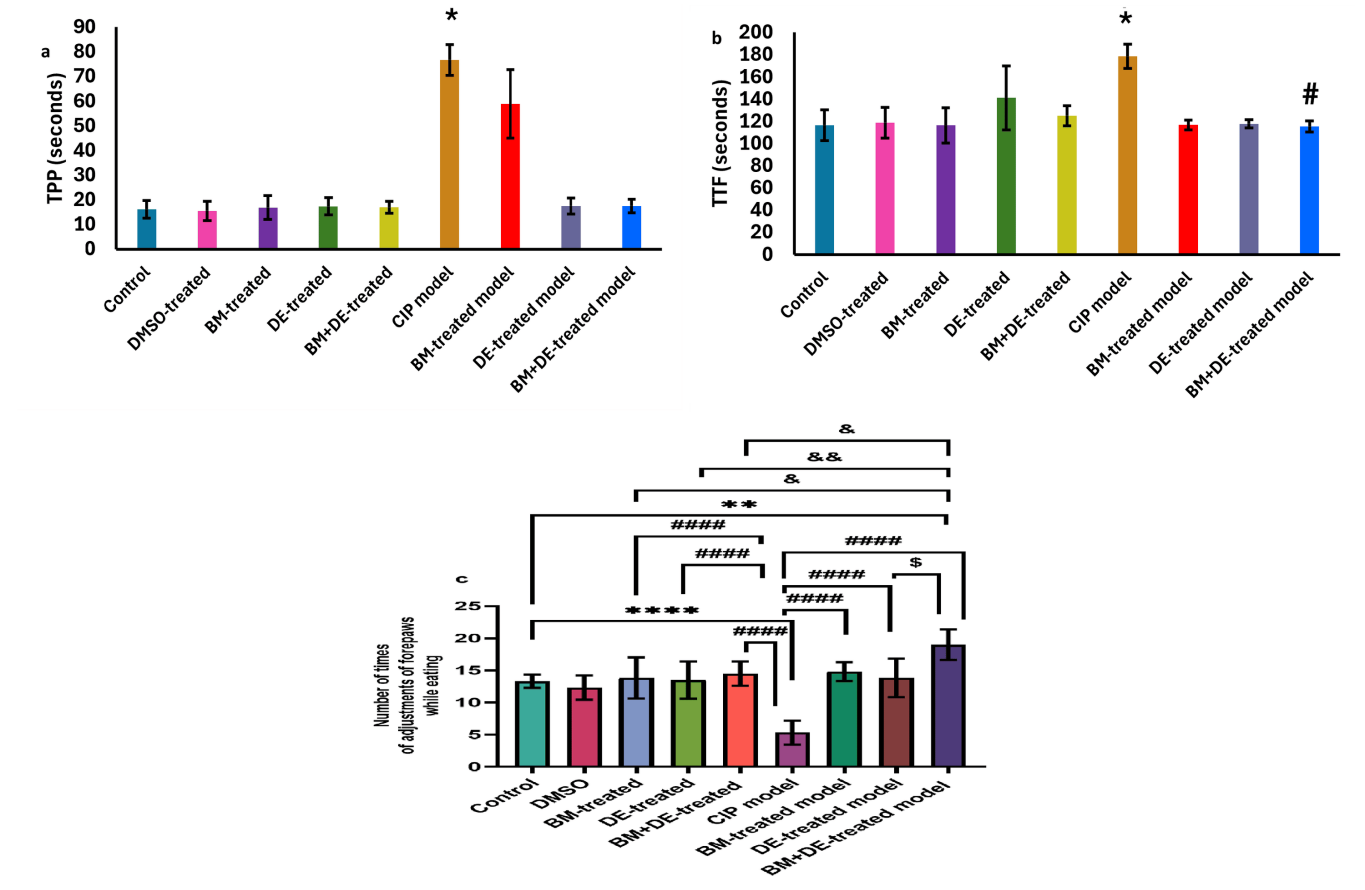
BM+DE improved pasta handling (n=6). a) Time to pick up pasta from the cage floor; b) Time to finish eating; c) Number of times of adjustments of forepaws while eating. Data are expressed as mean ± SD. DMSO: dimethyl sulfoxide; CIP: cobalt chloride-induced parkinsonism; BM: benztropine mesylate; DE: dabigatran etexilate. *Ρ < 0.05, **Ρ < 0.01, ****Ρ < 0.0001 vs controls; # Ρ < 0.05, # # # # Ρ < 0.0001 vs CIP; & Ρ < 0.05, & & Ρ < 0.01 vs BM+DE-treated model; $Ρ < 0.05 vs DE-treated model.

CoCl_2_ delayed the TPP relative to controls ((76.67±6.19 vs. 16.17±3.60 s) (t (9) = 29.20, Ρ < 0.05)) (Figure 3a), prolonged the TTF ((178.50±11.01 vs. control 116.50±13.98) (t (9) = 22.32, Ρ < 0.05)) (Figure 3b), and reduced the NTAF ((5.33±1.86 vs. 13.33±1.03) (F (8,45) = 14.63, Ρ < 0.0001)) (Figure 3c). Unlike BM and DE, which did not alter TPP or TTF, BM+DE substantially improved the TTF, but not the TPP, to be accelerated relative to the CIP model ((115.50±4.97 vs. 178.50±11.01) (t (9) = 22.32, Ρ < 0.05)), restoring it to control level (Figure 3b). Nonetheless, BM and DE did not exhibit significant differences relative to controls regarding TPP or TTF. BM, DE, and BM + DE significantly increased the NTAF relative to the CIP model ((14.83±1.47, 13.83±2.99, and 19.00±2.37, respectively, vs. 5.33±1.86) (F (8,45) = 14.63, Ρ < 0.0001)). Both BM and DE restored the NTAF to the control level, while the NTAF of BM+DE-treated CIP rats significantly exceeded the control level ((13.33±1.03) (F (8,45) = 14.63, Ρ < 0.01)) (Figure 3c).

DE combined with BM restored DA and D2 to control levels

**Figure 4:**
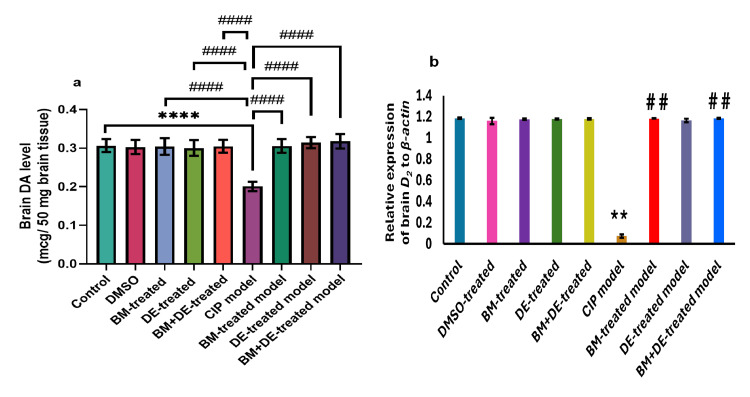
BM+DE restored brain dopaminergic signaling (n=3). a) Dopamine (DA); b) Dopamine receptor type 2 (D2). Data are expressed as mean ± SD. DMSO: dimethyl sulfoxide; CoCl_2_; cobalt chloride; CIP: cobalt chloride-induced parkinsonism; BM: benztropine mesylate; DE: dabigatran etexilate. **Ρ < 0.01, ****Ρ < 0.0001 vs controls; # #Ρ < 0.01, # # # #Ρ < 0.0001 vs CIP.

Brain DA level was reduced in the CIP model relative to controls ((0.20±0.01 against 0.31±0.02) (F (8,45) = 24.63, Ρ < 0.0001)) (Figure 4a). Likewise, brain D2 mRNA expression in the CIP model was downregulated relative to controls ((0.08±0.02 vs. 1.19±0.01) (t (9) = 28.12, Ρ < 0.01)) (Figure 4b). Relative to the untreated CIP model, BM, DE, and BM+DE significantly increased brain DA ((0.31±0.02, 0.32±0.02, and 0.32±0.02, respectively, vs. 0.20±0.01) (F (8,45) = 24.63, Ρ < 0.0001)), restoring it to control levels. Notably, no significant differences were found comparing the three treated model groups (Figure 4a). Both BM and BM+DE significantly upregulated D2 ((1.18±0.00 and 1.18±0.01, respectively, vs. 0.08±0.02) (t (9) = 28.12, Ρ < 0.01)), restoring it to control levels; however, DE did not cause a significant effect relative to untreated CIP model or controls (Figure 4b).

The brain level of DA was positively correlated with the relative expression of brain D2 (r = 0.319, Ρ <0.05). Correlations between dopaminergic signaling and behavioral indices are illustrated in Figure 11 (Appendices).

DE combined with BM restored A1 and A2A mRNA to control levels, yielding an A1/A2A expression ratio exceeding the control level

**Figure 5:**
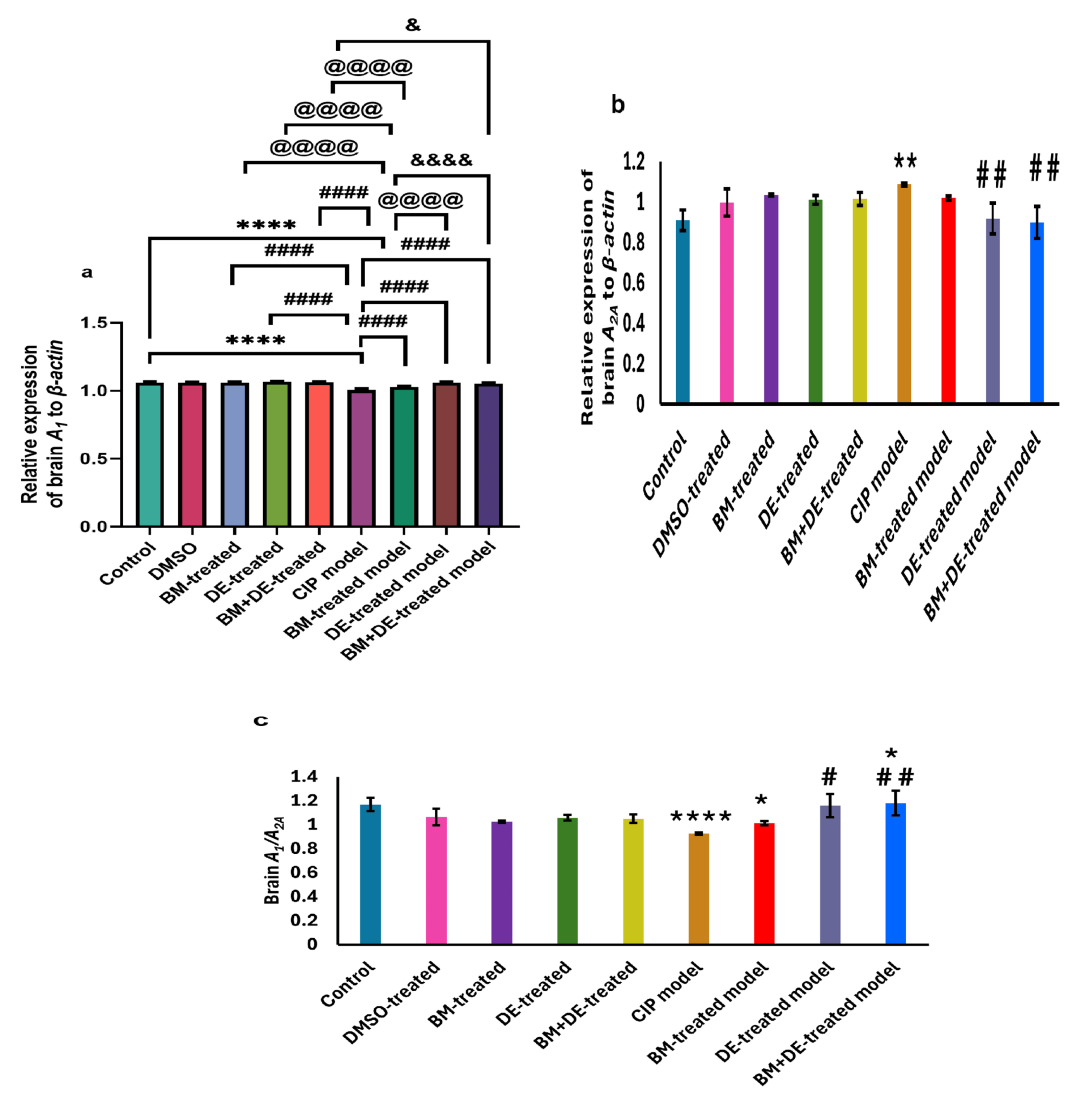
BM+DE restored brain adenosinergic signaling (n=3). a) Adenosine receptor type 1 (A1); b) Adenosine receptor type 2A (A2A); c) Ratio of A1/A2A. Data are expressed as mean ± SD. DMSO: dimethyl sulfoxide; CIP: cobalt chloride-induced parkinsonism; BM: benztropine mesylate; DE: dabigatran etexilate *Ρ < 0.05, **Ρ < 0.01, ****Ρ < 0.0001 vs controls; #Ρ < 0.05, # #Ρ < 0.01, # # # #Ρ < 0.0001 vs CIP; @ @ @ @Ρ < 0.0001 vs BM-treated model; &Ρ < 0.05, &&&&Ρ < 0.0001 vs BM+DE-treated model.

The CIP model showed significant downregulation of brain A1 expression relative to controls ((1.01±0.01 vs. 1.06±0.01) (F (8, 45) = 56.60, Ρ < 0.0001)) (Figure 5a) and a significant upregulation of brain A2A ((1.09±0.01 vs. 0.91±0.05) (t (9) = 32.90, Ρ < 0.01)) (Figure 5b). These CoCl_2_-mediated changes yielded a significantly reduced A1/A2A ratio ((0.93±0.01 vs. 1.17±0.06) (t (9) = 35.56, Ρ < 0.0001)) (Figure 5c). Relative to the untreated CIP model, BM, DE, and BM+DE upregulated brain A1 expression ((1.03±0.01, 1.06±0.01, and 1.05±0.01, respectively) (F (8, 45) = 56.60, Ρ < 0.0001)). In this context, the effects of both DE and BM+DE exceeded that of BM (F (8, 45) = 56.60, Ρ < 0.0001), such that brain A1 expression remained significantly lower in the BM-treated model group relative to controls (F (8, 45) = 56.60, Ρ < 0.0001) (Figure 5a). As for brain A2A expression, it was downregulated by both DE and BM+DE relative to the CIP model ((0.90±0.08) (t (9) = 32.90, Ρ < 0.01)), restoring it to control levels (Figure 5b). This was not the case with BM which did not alter brain A2A expression relative to the CIP model, yet with no significant difference compared to controls. Treating the CIP model using DE and BM+DE increased the A1/A2A ratio ((1.16±0.10 and 1.18±0.10, respectively) (t (9) = 35.56, Ρ < 0.05, (t (8) = 35.56, Ρ < 0.01, respectively)). The increased brain A1/A2 ratio was more substantial with BM+DE as evidenced by a significantly higher level relative to controls (t (9) = 35.56, Ρ < 0.05). BM did not achieve a significant effect on the brain A1/A2A ratio which remained significantly reduced relative to controls ((1.01±0.02) (t (9) = 35.56, Ρ < 0.05)) (Figure 5c).

Notably, the relative expression of brain A2A was negatively correlated with brain DA levels (r = -0.547, Ρ <0.0001) as well as with brain D2 expression (r = -0.303, Ρ <0.05). A1/A2A ratio was positively correlated with DA levels (r = 0.513, Ρ <0.0001). These correlations are illustrated in Figure 12 (Appendices).

Correlations of adenosinergic signaling with motor behavior

A2A expression was negatively correlated with the number of rears (r = -0.528, Ρ <0.0001) as well as with the average displacement distance of the right forepaw (r = -0.522, Ρ <0.0001). In contrast, the A1/A2A ratio was positively correlated with the average displacement distance of the right forepaw (r = 0.510, Ρ <0.0001). Brain A1 expression was negatively correlated with the TPP (r = -0.546, Ρ <0.0001). These and other correlations are illustrated in Figure 13 (Appendices).

DE combined with BM restored PAR1 expression and ET1 to control levels

**Figure 6:**
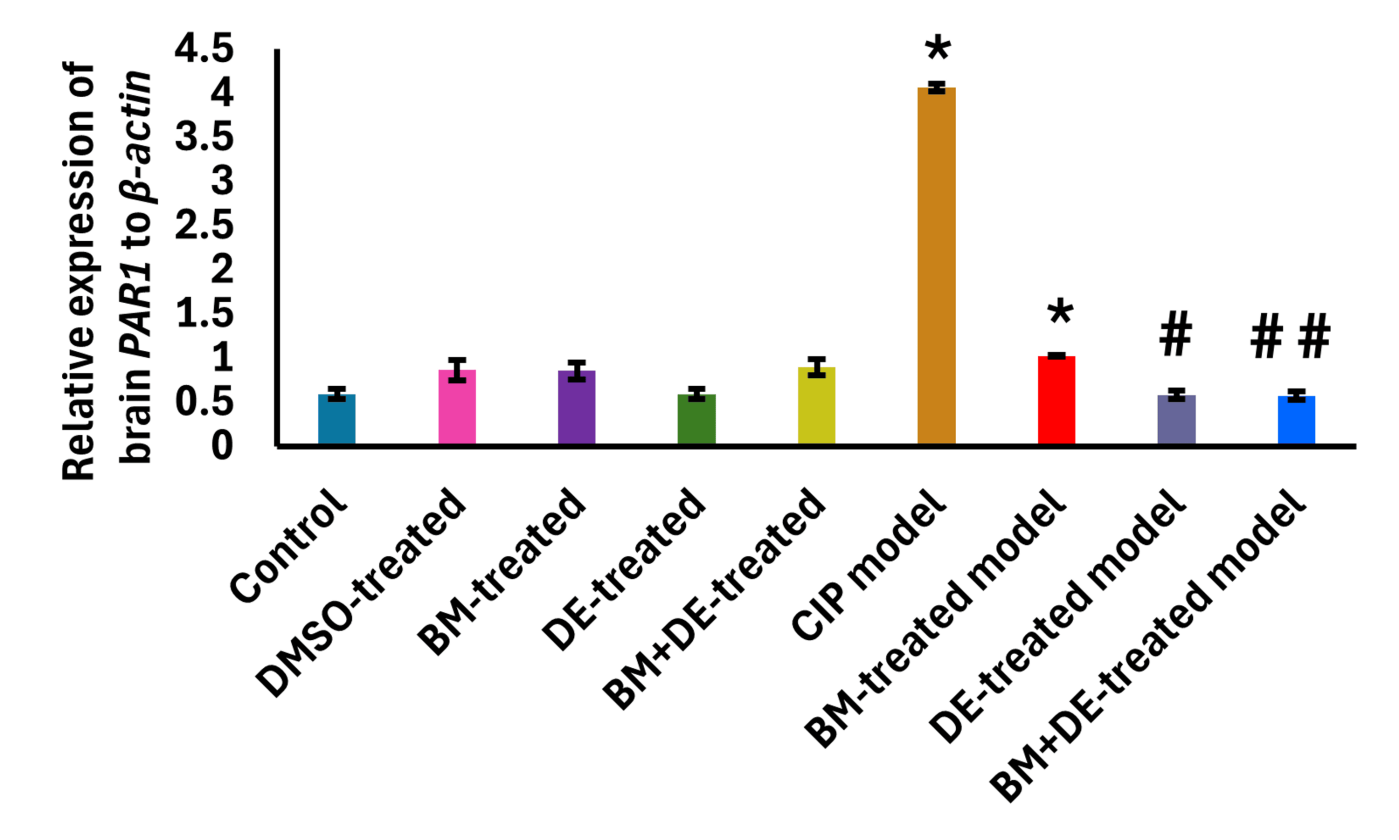
BM+DE restored brain protease-activated receptor 1 (PAR1) expression (n=3). Data are expressed as mean ± SD. DMSO: dimethyl sulfoxide; CIP: cobalt chloride-induced parkinsonism; BM: benztropine mesylate; DE: dabigatran etexilate *Ρ < 0.05 vs controls; #Ρ < 0.05, # #Ρ < 0.01 vs CIP

**Figure 7:**
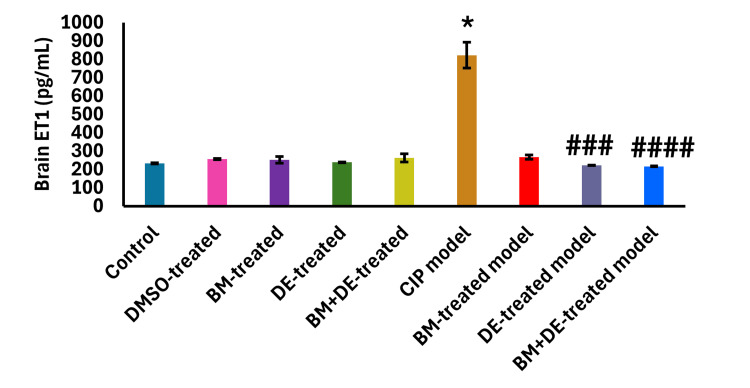
BM+DE restored brain endothelin 1 (ET1) level (n=3). Data are expressed as mean ± SD. DMSO: dimethyl sulfoxide; CIP: cobalt chloride-induced parkinsonism; BM: benztropine mesylate; DE: dabigatran etexilate *Ρ < 0.05 vs controls; ###Ρ < 0.001, ####Ρ < 0.0001 vs CIP.

The CIP model significantly upregulated brain PAR1 expression compared to control levels ((4.06±0.04 vs. 0.79±0.15) (t (9) = 46.47, Ρ < 0.05)) (Figure 6), together with increasing brain ET1 levels ((821.64±70.38 against 233.5±3.16) (t (9) = 44.48, Ρ < 0.05)) (Figure 7). Both DE and BM+DE downregulated brain PAR1 expression compared to the untreated CIP model ((0.76±0.16 and 0.72±0.11, respectively) (t (9) = 46.47, Ρ < 0.05, t (9) = 46.47, Ρ < 0.01, respectively)), restoring it to control levels. In contrast, BM did not significantly affect brain PAR1 expression which remained significantly higher than controls ((1.02±0.01) (t (9) = 46.47, Ρ < 0.05)) (Figure 7). Both DE and BM+DE reduced brain ET1 levels compared to the untreated CIP model ((222.14±1.42 and 216.32±2.41, respectively) (t (9) = 44.48, Ρ < 0.001, t (9) = 44.48, Ρ < 0.0001, respectively)), restoring it to control levels. This was not the case with BM which did not significantly affect brain ET1, yet with no significant variation from the controls (Figure 7). The brain level of ET1 was positively correlated with the relative expression of brain PAR1 (r = 0.812, Ρ <0.0001).

Correlations of the thrombin-related factors to motor behavior and dopaminergic and adenosinergic signaling

Both PAR1 expression and ET1 levels were negatively correlated with the number of rears (r = -0.594, Ρ <0.0001; r = -0.528, Ρ <0.0001, respectively). These and other correlations are illustrated in Figure 14 (Appendices). Both PAR1 expression and ET1 levels were positively correlated with A2A expression (r = 0.571, Ρ <0.0001; r = 0.582, Ρ <0.0001, respectively). Conversely, both PAR1 and ET1 were negatively correlated with A1/A2A ratio (r = -0.669, Ρ < 0.0001; r = -0.648, Ρ < 0.0001, respectively). These and other correlations are illustrated in Figures 15-16 (Appendices).

DE combined with BM restored MDA and MDA/GSH ratio and increased GSH beyond control levels

**Figure 8:**
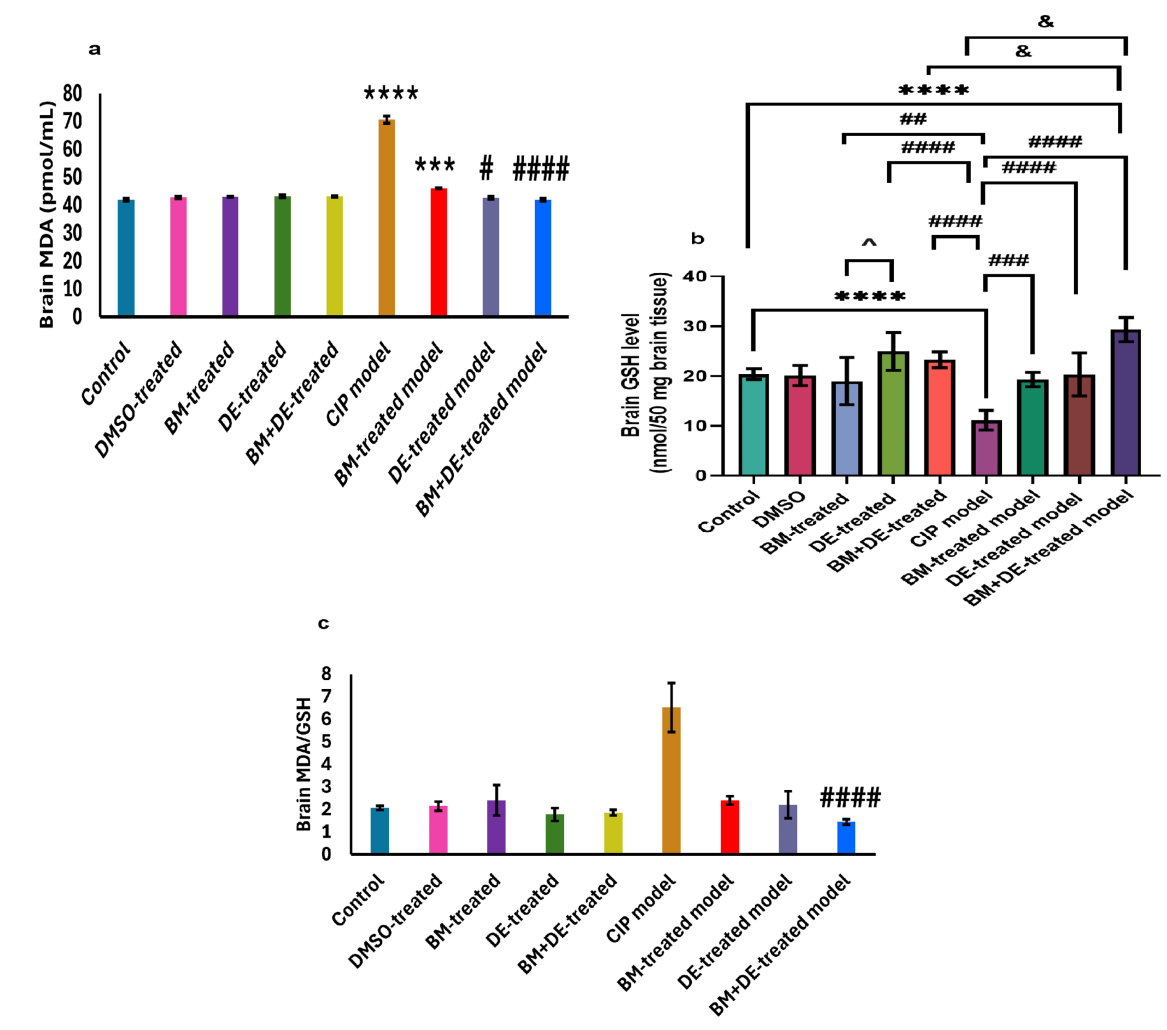
BM+DE exerted an antioxidant effect (n=3). a) Malondialdehyde (MDA); b) Glutathione (GSH); c) Ratio of MDA/GSH. Data are expressed as mean ± SD. DMSO: dimethyl sulfoxide; CIP: cobalt chloride-induced parkinsonism; BM: benztropine mesylate; DE: dabigatran etexilate *** Ρ < 0.001, ****Ρ < 0.0001 vs controls; # Ρ < 0.05, ## Ρ < 0.01, ### Ρ < 0.001, ####Ρ < 0.0001 vs CIP; &Ρ < 0.05 vs BM+DE-treated model; ^ Ρ < 0.05 vs BM-treated control.

A pronounced increase in brain MDA level was observed in the CIP model compared to controls ((70.62±1.28 vs. 41.93±0.39) (t (9) = 44.97, Ρ <0.0001)) (Figure 8a) against a prominent reduction in brain GSH level ((11.11±2.00 vs. 20.39±1.07) (F (8, 45) = 17.63, Ρ <0.0001)) (Figure 8b). Nonetheless, MDA/GSH did not show any significant change. Both DE and BM+DE significantly reduced brain MDA levels relative to the CIP model ((42.64±0.44 and 42.01±0.47, respectively) (t (9) = 44.97, Ρ <0.05, t (9) = 44.97, Ρ <0.0001, respectively)), restoring it to the control level. In contrast, BM did not significantly modify brain MDA level (46.02±0.05) which remained significantly higher than controls (t (9) = 44.97, Ρ <0.01) (Figure 8a). BM, DE, and BM+DE significantly increased brain GSH levels relative to the CIP model ((19.31±1.42, 20.30±4.33 and 29.33±2.43, respectively) (F (8, 45) = 17.63, Ρ <0.001, F (8, 45) = 17.63, Ρ <0.0001, and F (8, 45) = 17.63, Ρ <0.0001, respectively). BM+DE exhibited the highest effect on brain GSH level, exceeding the control level (F (8, 45) = 17.63, Ρ <0.0001. This was not the case with either BM or DE, in which GSH was restored to the control level (Figure 8b). Also, BM+DE significantly reduced brain MDA/GSH (1.44±0.11) compared to the untreated CIP model (6.52±1.09) (t (9) = 38.33, Ρ <0.0001) (Figure 8c). Notably, the brain level of MDA was negatively correlated with that of GSH (r = -0.445, Ρ <0.001).

Correlations of oxidative stress with motor behavior as well as adenosinergic, and thrombin-related biomarkers

MDA level was negatively correlated with the number of rears (r = -0.602, Ρ <0.0001) and the average displacement distance of the right forepaw (r = -0.563, Ρ <0.0001). Conversely, the GSH level was positively correlated with the average displacement distance of the left forepaw (r = 0.512, Ρ <0.001) and the NTAF (r = 0.600, Ρ <0.0001). Unlike GSH, the MDA/GSH ratio was negatively correlated with the average displacement distance of the left forepaw (r = -0.505, Ρ <0.001). and with the NTAF (r = -0.575, Ρ <0.0001). These and other correlations are illustrated in Figure 17 (Appendices). MDA was positively correlated with A2A (r = 0.651, Ρ <0.0001) while being negatively correlated with A1/A2A ratio (r = -0.732, Ρ <0.0001). Moreover, MDA was positively correlated with both PAR1 and ET1 (r = 0.726, Ρ <0.0001; r = 0.748, Ρ <0.0001, respectively). Unlike MDA, GSH was negatively correlated with ET1 (r = -0.573, Ρ <0.0001). MDA/GSH ratio was negatively correlated with A1 (r = -0.510, Ρ <0.001). Conversely, MDA/GSH was positively correlated with both PAR1 and ET1 (r = 0.726, Ρ <0.0001; r = 0.748, Ρ <0.0001, respectively). These and other correlations are illustrated in Figures 18-20 (Appendices).

DE combined with BM partially restored the neurodegenerative changes induced by CoCl_2_

**Figure 9:**
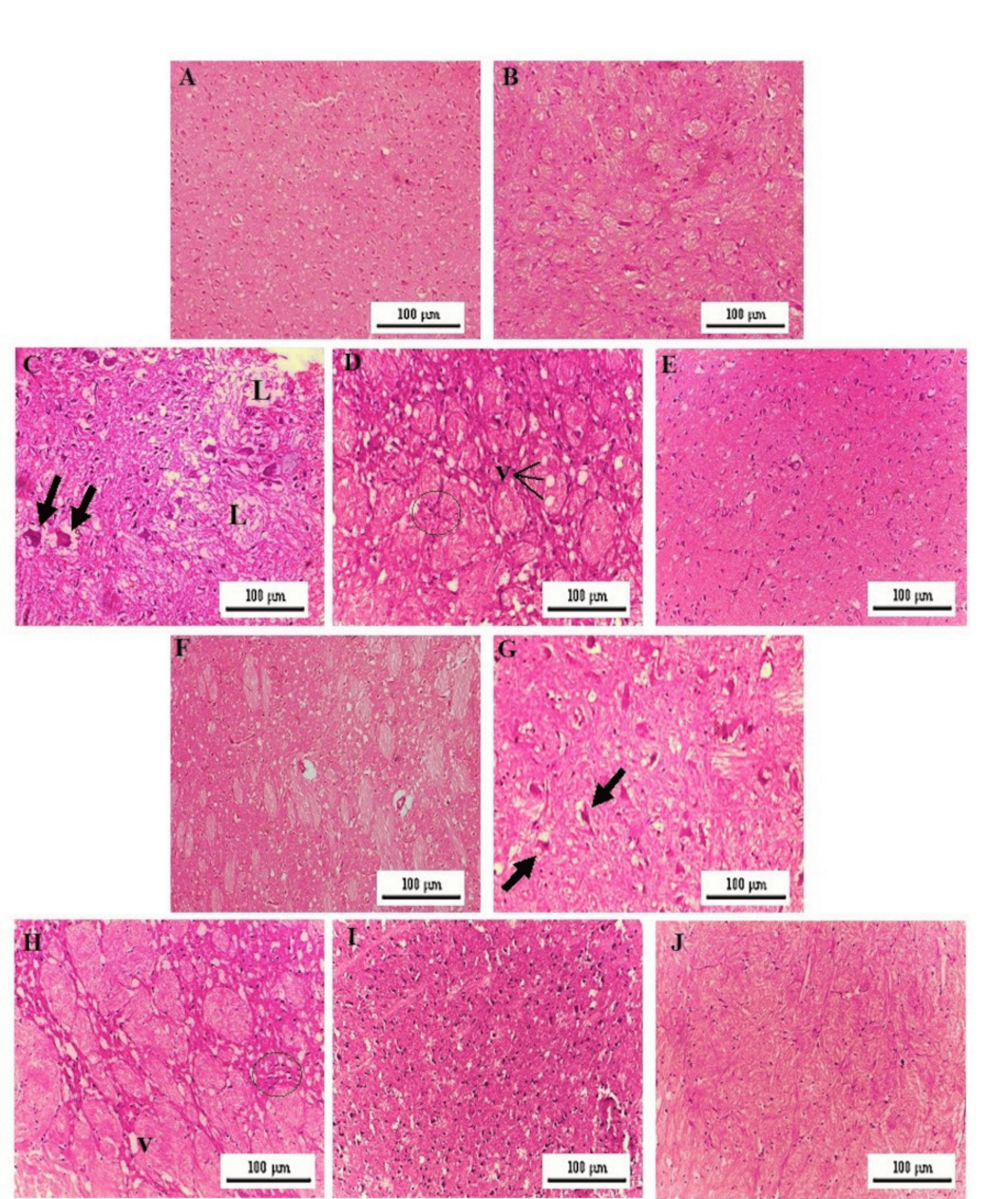
BM+DE mitigated neuroinflammatory and neurodegenerative changes (H&E-stained sections of substantia nigra (SN) and striatum (STR)). a) SN of controls, DMSO, BM-, DE-, and BM+DE- treated controls; b) STR of controls, DMSO, BM-, DE-, and BM+DE- treated controls; c) SN of CIP showing neuronal loss (L) and exhibiting Lewy bodies (arrows); d) STR of CIP revealing vacuolation (V) and inflammatory cellular infiltration (circle); e-f) Respective SN and STR of BM-treated model group displaying normal microstructure; g) SN of DE-treated model exhibiting Lewy bodies (arrows); h) STR of DE-treated model showing persistence of inflammatory infiltrate (circle) with scattered vacuolation (V); i-j) Respective SN and STR of BM+DE-treated model showing normal architecture. SN: substantia nigra; STR: striatum; DMSO: dimethyl sulfoxide; CIP: cobalt chloride-induced parkinsonism; BM: benztropine mesylate; DE: dabigatran etexilate

In contrast to the normal microstructure of the SN and STR of controls, BM-, DE-, and BM+DE-treated controls (Figures 9a-9b), the CIP model was afflicted by neuronal degeneration in the SN with evidence of Lewy bodies. Also, vacuolation as well as inflammatory infiltrates appeared within the STR (Figures 9c-9d). In the BM-treated model group, both the SN and STR had an apparently normal architecture (Figures 9e-9f). In the DE-treated model group, Lewy bodies persisted in the SN (Figure 9g), and inflammatory infiltrates as well as scattered vacuoles were found in the STR (Figure 9h). The BM+DE-treated model group exhibited normal SN (Figure 9i) and STR microstructures (Figure 9j).

DE combined with BM exhibited the highest percent of preserved neurons among the treated model groups

**Figure 10:**
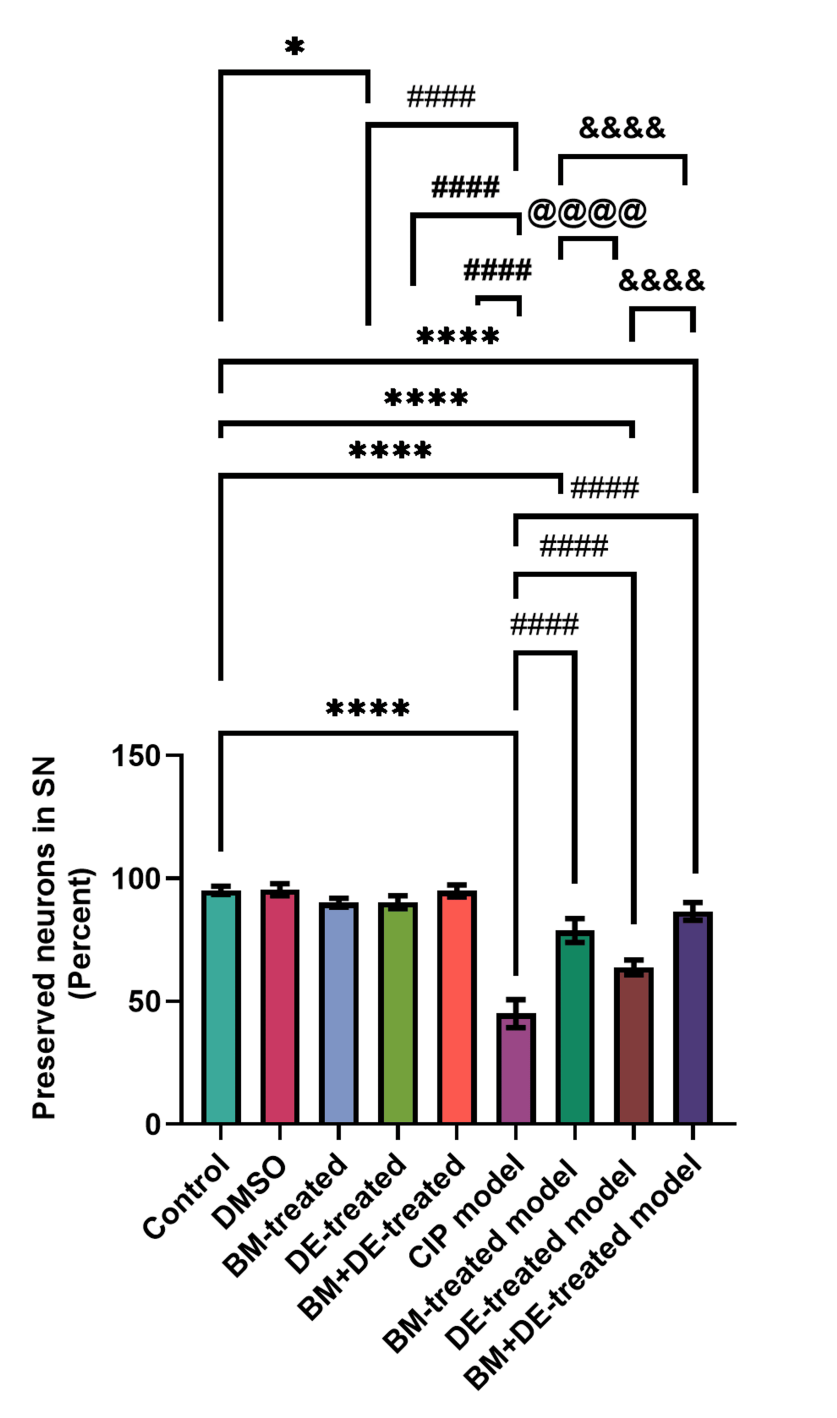
BM+DE partially restored dopaminergic neurons (n=3). Data are expressed as mean ± SD. SN: substantia nigra; STR: striatum; DMSO: dimethyl sulfoxide; CIP: cobalt chloride-induced parkinsonism; BM: benztropine mesylate; DE: dabigatran etexilate *Ρ < 0.05, ****Ρ < 0.0001 vs controls; # # # #Ρ < 0.0001 vs CIP model; @ @ @ @ Ρ < 0.0001 vs BM-treated model; &&&& Ρ < 0.0001 vs BM+DE-treated model.

**Figure 11:**
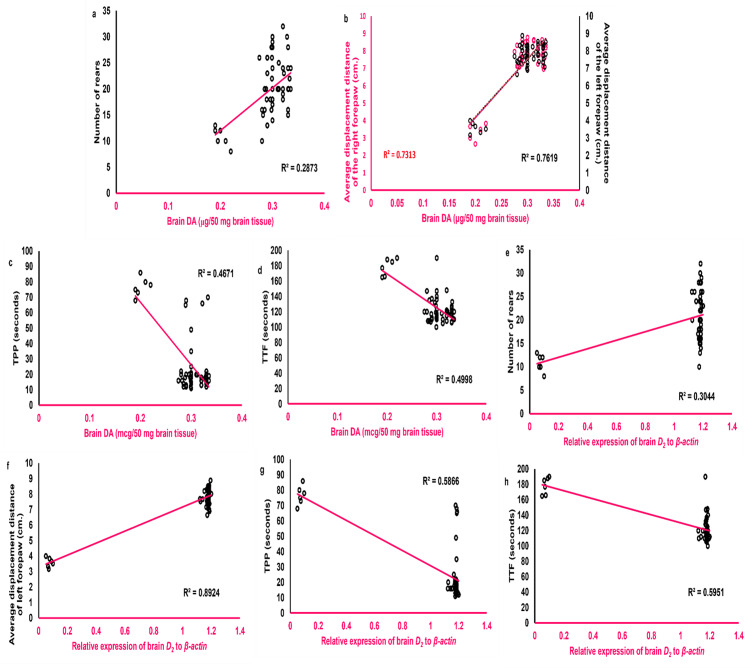
Significant correlations between dopaminergic signaling and motor indices. a) Dopamine (DA) and number of rears (r = 0.391, Ρ <0.01), b) DA and the average displacement distances of the right and left forepaws (r = 0.357, P <0.05; r = 0.457, Ρ <0.01, respectively), c) DA and time to pick pasta (TPP) (r = -0.293, Ρ <0.05), d) DA and time to finish eating (TTF) (r = -0.299, Ρ <0.05), e) Dopamine receptor type 2 (D2) and the number of rears (r = 0.400, Ρ <0.01), f) D2 and the average displacement distance of the left forepaw (r = 0.445, Ρ <0.01), g) D2 and TPP (r = -0.346, Ρ <0.05), h) D2 and TTF (r = -0.346, Ρ <0.05). DA: dopamine; D2: dopamine receptor type 2; TPP: time to pick pasta; TTF: time to finish eating Spearman rho. Significant when Ρ < 0.05.

**Figure 12:**
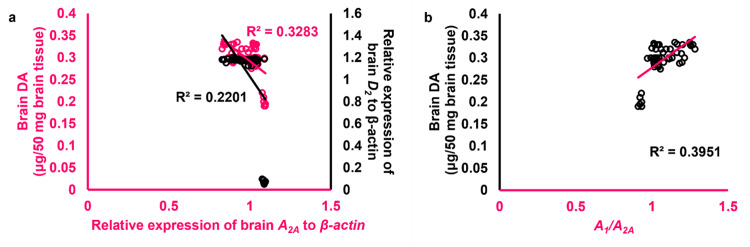
Significant correlations between dopaminergic and adenosinergic signaling. a) Adenosine receptor type 2A (A2A) and each of dopamine (DA) and dopamine receptor type 2 (D2), b) A1/A2A and DA. A2A: adenosine receptor type 2A, A1/A2A: ratio of adenosine receptor type 1 and adenosine receptor type 2A; DA: dopamine; D2: dopamine receptor type 2. Spearman rho. Significant when Ρ < 0.05.

**Figure 13:**
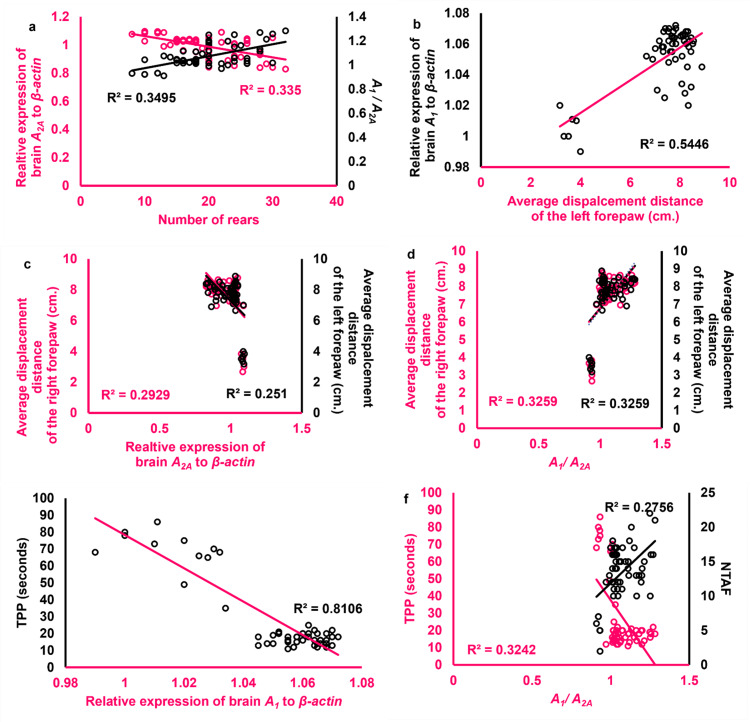
Significant correlations between adenosinergic signaling and motor indices. a) each of adenosine receptor type 2A (A2A) and adenosine receptor type 1 (A1)/A2A, and number of rears, b) A1 and the average displacement distance of the left forepaw, c) A2A and the average displacement distance of each of the right and the left forepaw, d) A1/A2A and the average displacement distance of each of the right and the left forepaw, e) A1 and time to pick pasta (TPP), f) A1/A2A and each of TPP and number of times of adjustments of forepaws (NTAF). A1: adenosine receptor type 1; A2A: adenosine receptor type 2A; A1/A2A: ratio of adenosine receptor type 1 and adenosine receptor type 2A; TPP: time to pick up pasta; NTAF: number of times of adjustment of forepaws. Spearman rho.  Significant when Ρ < 0.05.

**Figure 14:**
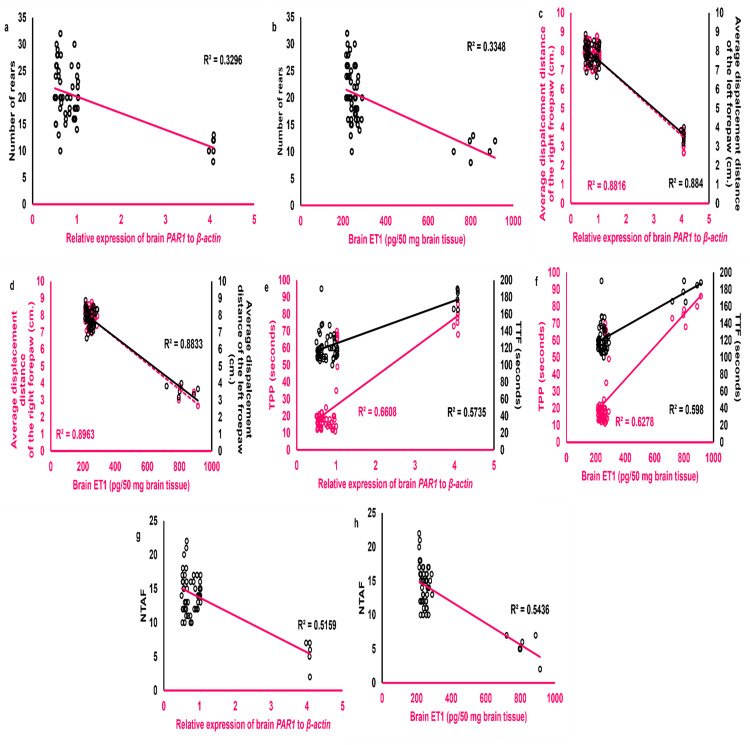
Significant correlations among protease-activated receptor 1 (PAR1), endothelin 1 (ET1), and motor indices. a) Protease-activated receptor 1 (PAR1) and the number of rears, b) Endothelin 1 (ET1) and the number of rears, c) PAR1 and the average displacement distance of each of the right and the left forepaw, d) ET1 and the average displacement distance of each of the right and the left forepaw, e) PAR1 and each of time to pick pasta (TPP) and time to finish eating (TTF), f) ET1 and each of TPP and TTF, g) PAR1 and number of times of adjustments of forepaws (NTAF), h) ET1 and NTAF. PAR1: protease-activated receptor 1; ET1: endothelin 1; TPP: time to pick up pasta; TTF: time to finish eating; NTAF: number of times of adjustment of forepaws. Spearman rho. Significant when Ρ < 0.05.

**Figure 15:**
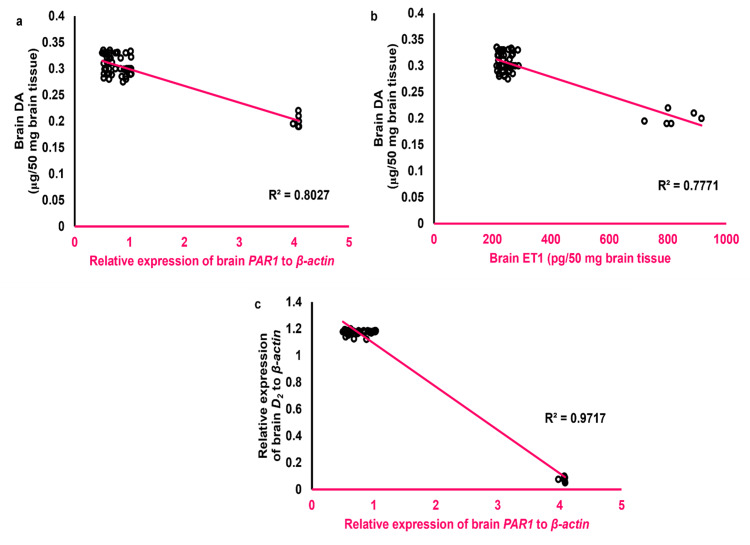
Significant correlations between dopaminergic signaling and each of protease-activated receptor 1 (PAR1) and endothelin 1 (ET1). a) Protease-activated receptor 1 (PAR1) and dopamine (DA), b) Endothelin 1 (ET1) and DA, c) PAR1 and dopamine receptor type 2 (D2). PAR1: protease-activated receptor 1; ET1: endothelin 1; DA: dopamine; D2: dopamine receptor type 2 Spearman rho. Significant when Ρ < 0.05.

**Figure 16:**
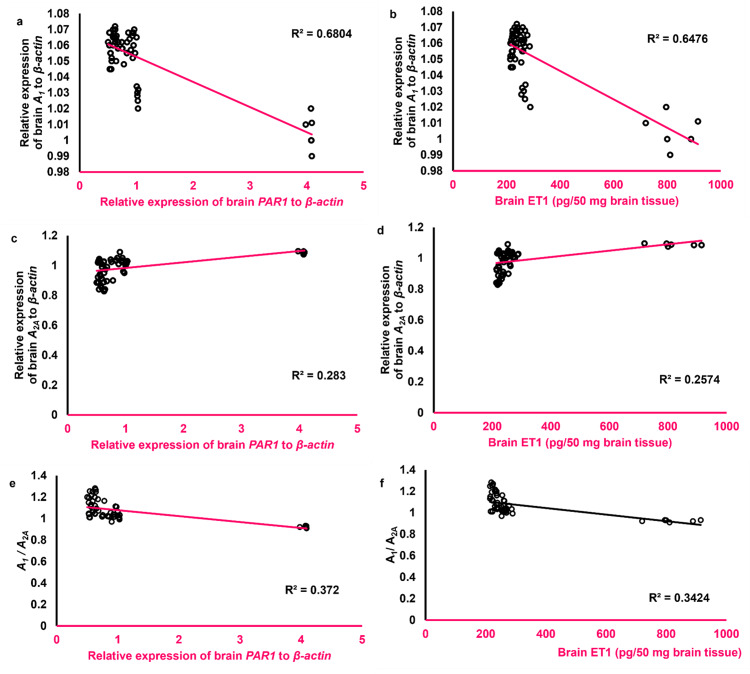
Significant correlations between adenosinergic signaling and each of protease-activated receptor 1 (PAR1) and endothelin 1 (ET1). a) Protease-activated receptor 1 (PAR1) and adenosine receptor type 1 (A1), b) Endothelin 1 (ET1) and A1, c) PAR1 and adenosine receptor type 2A (A2A), d) ET1 and A2A, e) PAR1 and A1/A2A, f) ET1 and A1/A2A. PAR1: protease-activated receptor 1; ET1: endothelin 1; A1: adenosine receptor type 1; A2A: adenosine receptor type 2A; A1/A2A: ratio of adenosine receptor type 1 and adenosine receptor type 2. Spearman rho.  Significant when Ρ < 0.05.

**Figure 17:**
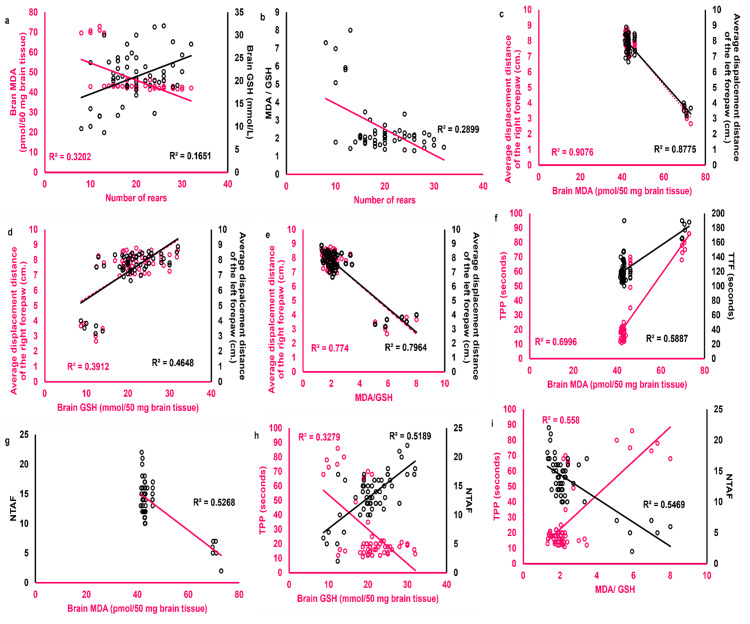
Significant correlations between motor indices and each of malondialdehyde (MDA) and glutathione (GSH). a) each of MDA and GSH, and the number of rears, b) MDA/GSH and the number of rears, c) MDA and the average displacement distance of each of the right and the left forepaw, d) GSH and the average displacement distance of each of the right and the left forepaw, e) MDA/GSH and the average displacement distance of each of the right and the left forepaw, f) MDA and each of TPP and TTF, g) MDA and NTAF, h) GSH and each of TPP and NTAF, i) MDA/GSH and each of TPP and NTAF. MDA: malondialdehyde; GSH: reduced glutathione; MDA/GSH: ratio of malondialdehyde and reduced glutathione; TPP: time to pick up pasta; TTF: time to finish eating; NTAF: number of times of adjustment of forepaws. Spearman rho.  Significant when Ρ < 0.05.

**Figure 18:**
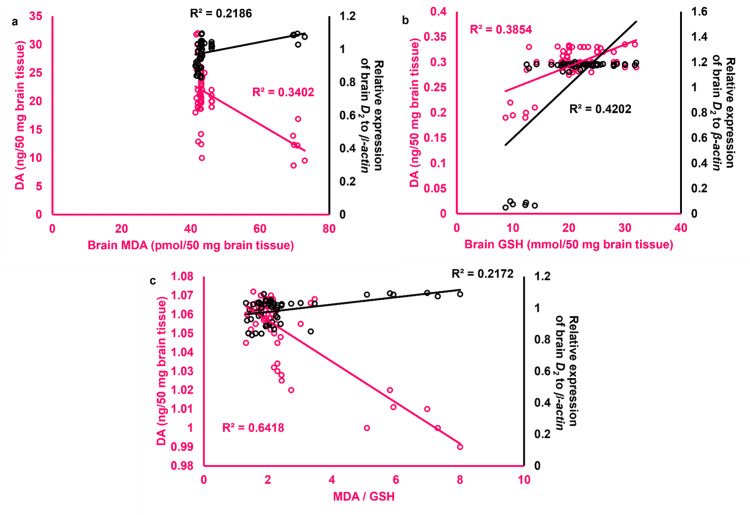
Significant correlations between dopaminergic signaling and each of, malondialdehyde (MDA) and glutathione (GSH). a) Malondialdehyde (MDA) and each of dopamine (DA) and dopamine receptor type 2 (D2), b) Glutathione (GSH) and each of DA and D2, c) MDA/GSH and each of DA and D2. MDA: malondialdehyde; GSH: reduced glutathione; MDA/GHS: ratio of malondialdehyde and reduced glutathione; DA: dopamine; D2: dopamine receptor type 2. Spearman rho. Significant when Ρ < 0.05.

**Figure 19:**
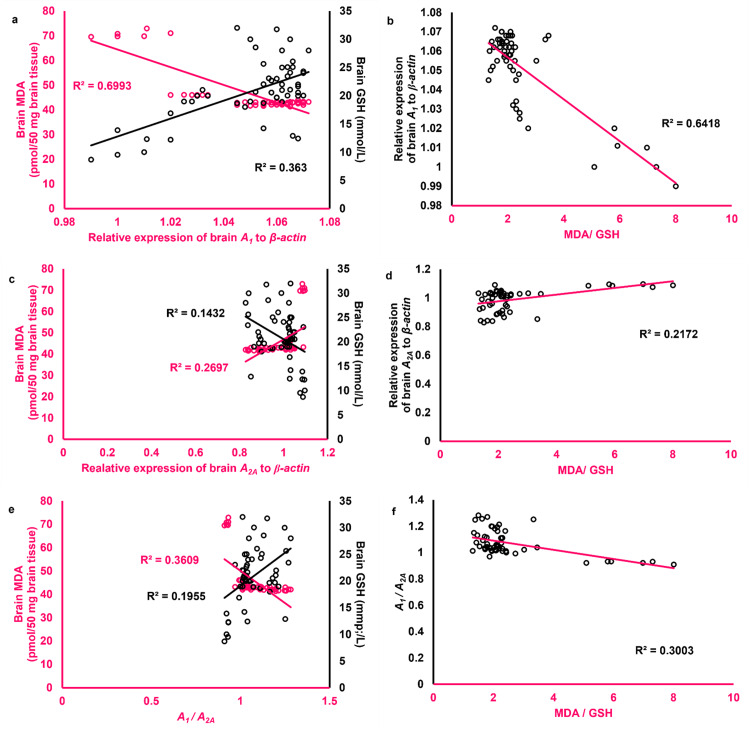
Significant correlations between adenosinergic signaling and each of, malondialdehyde (MDA) and glutathione (GSH). a) each of malondialdehyde (MDA) and glutathione (GSH), and adenosine receptor type 1 (A1), b) MDA/GHS and A1, c) each of MDA and GSH, and adenosine receptor type 2A (A2A), d) MDA/GSH and A2A, e) each of MDA and GSH, and A1/A2A, f) MDA/GSH and A1/A2A. MDA: malondialdehyde; GSH: reduced glutathione; MDA/GSH: ratio of malondialdehyde and reduced glutathione; A1: adenosine receptor type 1; A2A: adenosine receptor type 2A; A1/A2A: ratio of adenosine receptor type 1 and adenosine receptor type 2. Spearman rho.  Significant when Ρ < 0.05.

**Figure 20:**
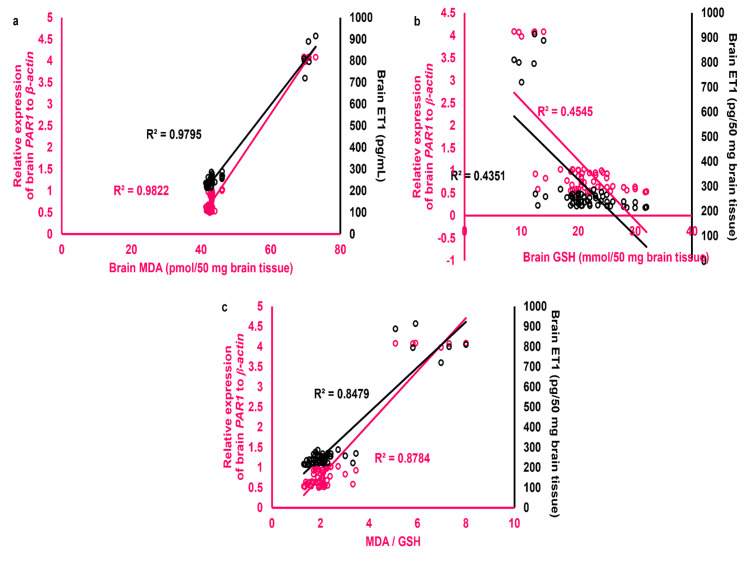
Significant correlations among protease-activated receptor 1 (PAR1), endothelin 1 (ET1), and each of malondialdehyde (MDA) and glutathione (GSH). a) Malondialdehyde (MDA) and each of protease-activated receptor 1 (PAR1) and endothelin 1 (ET1), b) Glutathione (GSH) and each of PAR1 and ET1, c) MDA/GSH and each of PAR1 and ET1. MDA: malondialdehyde; GSH: reduced glutathione; MDA/GSH: ratio of malondialdehyde and reduced glutathione; PAR1: protease-activated receptor 1; ET1: endothelin 1. Spearman rho.  Significant when Ρ < 0.05.

Remarkably, BM-treated controls showed a significantly increased percentage of preserved neurons in the SN (F (8, 81) = 259.80, Ρ < 0.05). This was not the case with DE or BM+DE-treated controls. The CIP model exhibited a significantly reduced percentage of preserved neurons in the SN relative to controls ((45.00±5.66 against 95.10 ±1.66) (F (8,81) = 259.80, Ρ < 0.0001)) (Figure 10). The percentage of preserved neurons in the SN was significantly higher in the BM, DE, and BM+DE-treated CIP rats (78.80±4.87, 63.80±3.05 and 86.60±3.63, respectively) than that of the untreated CIP model rats (F (8, 81) = 259.80, Ρ < 0.0001); however, the percentage of preserved neurons in the SN remained significantly lower than controls (F (8, 81) = 259.80, Ρ < 0.0001). Notably, the percentage of preserved neurons in the SN with BM+DE treatment significantly exceeded those observed with BM and DE (F (8, 81) = 259.80, Ρ < 0.0001). Also, BM-treated CIP rats exhibited a significantly higher percentage of preserved neurons in the SN than DE-treated CIP rats (F (8, 81) = 259.80, Ρ < 0.0001) (Figure 10). Interestingly, the percent of preserved neurons in the SN was negatively correlated with brain MDA level (r = -0.285, Ρ <0.05).

## Discussion

Given the treatment-resistant cases of parkinsonism, short-lived therapeutic effects of some of the conventional antiparkinsonian medications, and non-compliance due to related side effects, there is increased interest in the exploration of novel antiparkinsonian modalities. Since patients with parkinsonism have a higher rate of thrombus formation [42]. DE was an attractive pharmacologic tool for exploration. The DE dose employed herein (3 mg/kg) was lower than the doses used in many hematologic studies in rats [43-45] because the central effects of thrombin are independent of its systemic procoagulant activity. In addition, the thrombin concentration needed to activate brain receptors is lower than that required for the conventional coagulation cascade [46].

In this study, the CIP model disturbed rearing behavior, mimicking the occurrence of a late, moderate-to-severe form of parkinsonism [47]. In partial agreement with our model, short-term (7 days) exposure to a higher dose of CoCl_2_ (150 mg/kg) precipitated motor incoordination in young adult male Wistar rats, associated with increased MDA and reduced GSH levels [48]. Compared to our CIP model, a 60-day low-dose rotenone administration to rodents did not cause prominent motor dysfunction [49]. A higher rotenone dose precipitated defective rearing behavior, yet caused mortality in the tested animals, with ill-defined pathological features [36], which was not the case in our model. Thus, the CIP model could offer a better alternative to other models in terms of survival on long-term exposure, while featuring a pronounced motor dysfunction and the pathognomonic neurodegenerative changes of parkinsonism. CoCl_2_-impaired motor performance in the pasta handling test, a test corresponding to impaired human reach-to-grasp behavior. The significance of the pasta handling paradigm relies on motor coordination programming. Motor incoordination was previously linked to dopaminergic deficit [50]. The CoCl_2_-associated motor dysfunction during the PIT highlighted the loss of postural reflexes. An atypical form of parkinsonism is anticipated if postural instability occurs within 3 years of onset, which resembles our CIP model [51].

DE, especially when combined with BM, produced substantial improvement in all three motor behavior assessments in this study, even exceeding the effect of BM at some point. In agreement with our results, in a rat model of rotenone-induced parkinsonism, oral DE delivered at five times the dose used in this study increased DA level [52]. Remarkably, the substantially higher preservation of dopaminergic neurons in the BM - than in the DE-treated model highlights the implication of non-dopaminergic mechanisms in motor improvement. Even though postural instability has been reported to occur with >80% dopamine depletion [53], the motor effect in our CIP model was obvious, despite less DA depletion and a mean preserved dopaminergic neurons in the SN of approximately 45%. Although a long period of CoCl_2_ was used to trigger neurotoxicity in this study, dopaminergic loss was not severe. Previous in-vivo studies demonstrated that exposure of both adult male Sprague-Dawley rats to 28-day IP CoCl_2_ (0.1, 0.0.5, or 1 mg/kg) and rabbits to 18-day intravenous CoCl_2_ injections triggered moderate neurotoxicity [54,55]. Paradoxical to the previously reported activation of presynaptic D2 autoreceptors to compensate for reduced DA, the positive correlation of DA level with D2 expression, identified in this study, indicates that this mechanism failed in the CIP model [56]. DE monotherapy failed to exert significant D2 upregulation relative to the untreated CIP model. Restoring DA to control levels with DE monotherapy is not a paradox though, given the partial enhancement of neurodegenerative changes. In this study, BM+DE therapy better restored SN and STR microstructures and dopaminergic neurotransmission than BM and DE monotherapies.

The correlation of motor behavior with dopaminergic signaling was not as strong as that with the non-dopaminergic pathways tested in this study. The CIP model might be considered a model of “atypical parkinsonism” in which the dopaminergic deficit occurs secondarily. In this case, poor response to levodopa and/or dopaminergic agonists is anticipated [57]. A reduced brain A1/A2A expression ratio, owing to A1 downregulation and A2A upregulation, was detected in the CIP model. A2A upregulation strongly correlated with some motor indices and showed a strong negative correlation to DA. A2A density was previously linked to the severity of parkinsonism and was negatively correlated to DA in postmortem patient specimens [58]. Meanwhile, in this study, the stronger correlation detected between the adenosinergic signaling and DA level (not D2 expression) suggested that A2A might regulate DA without heterodimerizing with D2. In this model, an A1 antagonist mitigated the potential therapeutic effect of dipyridamole, an antiplatelet and A2A antagonist, highlighting the implication of A1 in modulating responses to A2A [59]. The ability of BM, alone or combined with DE, to restore DA, D2, and A1, and to largely preserve dopaminergic neurons in SN, conflicts with other studies that found that A1 agonism counteracted the D2 agonistic activity [60,61]. Such discrepancies could be justified by the intricate A1/D2 interaction, as the local infusion of A1 antagonists inhibited DA release in some parts of the brain, whereas it enhanced DA release in other parts such as the nucleus accumbens [62]. Nonetheless, increasing A1/A2A with BM+DE treatment was consistent with the role of A1 as a neuroprotective factor during ischemia, hypoxia, and stroke [63], thus confronting A2A, regarded as a neurodegenerative factor in parkinsonism [64], and with the claims about A1 decreasing the potency and efficacy of A2A [65]. Studies addressing the disequilibration between A1 and A2A in parkinsonism are still lacking, a research gap that was addressed in our work. Although our study showed no significant correlations between A1 and either DA or D2, the strong positive correlation between DA and A1/A2A and the strong negative correlation between PAR1, ET1, and MDA, on one side, and A1/A2A, on the other side, highlighted the possibility that A1 can modulate DA by indirectly altering the optimum A1/A2A ratio, thus conferring antithrombotic, anti-inflammatory and antioxidant activities. The therapeutic efficacy of A1 agonists could be short-lived given that prolonged A1 activation induces its internalization, with a reported link to neuronal death by priming the brain for subsequent A2A-induced neurodegeneration [66]. For this reason, the therapeutic utility of a dual A1/A2A agonist/antagonist could be explored especially since A1/A2A was correlated with various motor indices in this study. In favor of this hypothesis, the best motor improvement was observed with BM+DE, when A1/A2A ratio exceeded the control level. In contrast to the persistently low A1/A2A ratio with BM administration, owing to a persistently lower A1 despite a restored A2A, DE monotherapy was able to restore the A1/A2A ratio. The notion that a non-selective adenosine antagonist such as caffeine was beneficial in patients with PD exhibiting postural instability and gait disturbance and the lack of significant correlation between A1 and A2A in our work highlighted that A1-A2A crosstalk is not sufficient to rationalize the changes observed in parkinsonism.

We assessed ET1 and PAR1, not only as indicators of vascular status of the brain but also relative to DE dynamics. The neurodegenerative and neuroinflammatory changes observed in the CIP model, along with PAR1 upregulation and ET1 increase, harmonizes with the work of Ishida et al. [21] who found increased PAR1 expression in postmortem specimens of patients with parkinsonism. The lack of significant correlation between PAR1 and the percentage of preserved neurons in the SN is in partial agreement with in vitro studies of rat neurons revealing that the thrombin-triggered dopaminergic neuronal loss in the SN is independent of PAR1 expression, unlike the thrombin-provoked hippocampal apoptosis that was mediated through PAR1 activation [67]. PAR1 restoration using DE, alone or combined with BM, was consistent with previous reports highlighting the ability of DE to attenuate PAR1 signaling comparable to PAR1 antagonists [68], subsequently reversing thrombin-triggered proinflammatory and pro-apoptotic activities [69]. Such changes matched the correlations between these thrombin-related factors and several behavioral indices. In contrast to our work, thrombin-activated PAR1 was shown to prevent PD-triggered motor dysfunction but with no beneficial effect on dopaminergic depletion [70]. Unlike DE, alone or combined with BM, BM did not significantly affect PAR1 in our CIP model. Some reports suggested that the addition of a thrombin inhibitor together with PAR1 manipulation could yield better outcomes, based on the ability of PAR1, in the presence of thrombin, to heterodimerize with other receptors, thus maintaining its functional activity [71]. Similarly, previous studies have highlighted the role of ET1 in systemic inflammation associated with parkinsonism [72-74]. Notably, the cascade of significant correlations between these thrombin-related factors and dopaminergic signaling, stronger when dealing with adenosinergic signaling, verified that adenosinergic signaling is an intermediary pathway by which thrombin-related signaling modulates dopaminergic neurotransmission. Increased brain levels of endothelin were previously associated with oxidative stress [75], which was corroborated in this study by the strong positive and negative correlations between ET1 and each of MDA and GSH, respectively. The oxidative stress-endothelin correlations were complemented by the strong positive correlations linking PAR1 to the MDA level and MDA/GSH ratio. Oxidative stress in parkinsonism was corroborated by the correlations between MDA and GSH, and their ratio, and multiple motor indices.

In line with the CIP-associated oxidative stress, adult male Wistar rats administered 20-day IP CoCl_2_ (4 mg/kg)-around one-third of the dose used herein-exhibited neuronal necrosis as well as elevated MDA in the hippocampus and temporal lobe [76]. Elevated MDA was also detected in patients with parkinsonism [15, 77]. In contrast, Ahlskog et al. and Çokal et al. reported that MDA did not differ in patients with PD, whether treated with levodopa or not [78-79]. BM+DE increased GSH beyond the control level while reducing MDA. DE monotherapy restored both MDA and GSH to control levels, while BM did not significantly affect MDA, which remained higher than the control level but was able to restore GSH to the control level. The ability of DE, alone or combined, to exert some antioxidant activity in the CIP model was previously explored using resveratrol [80], an agent that allows plasmin activation from its precursor, thus exerting a thrombolytic activity. The therapeutic benefit of increasing GSH beyond the control level with BM+DE was consistent with the mitigation of neurodegeneration in a previous rat model of parkinsonism when the antioxidant level was increased in dopaminergic neurons of SN [81]. The therapeutic utility of GSH in parkinsonism was justified in terms of the detoxification of byproducts of DA oxidation [82]. Nonetheless, the administration of oral GSH per se in parkinsonism was previously associated with modest efficacy [83]. Presumably, the antioxidant activity of BM+DE synergized its manipulation of the correlated adenosinergic and thrombin-related signaling, thus modulating the dopaminergic neurotransmission, culminating in a better preservation of dopaminergic neurons in SN and enhanced motor behavior. The significant negative correlation linking MDA, but not GSH, to the percentage of preserved neurons in SN, suggests a distinct role of antagonizing MDA to confer neuroprotective activity, which might apply to our CIP model, unlike other models of parkinsonism focusing on the prominent neuroprotective effects of GSH [84,85]. Targeting more than one pathway simultaneously could yield promising outcomes, as in our study.

Our work adopted the intricate pathways pertaining to parkinsonism, opening multiple alternative gateways to explore novel antiparkinsonian therapeutic modalities. To our knowledge, no previous study has addressed the multifaceted pathogenesis of parkinsonism in the CIP model, encompassing the dopaminergic, adenosinergic, thrombin-related, and oxidative stress signaling “tetralogy”.

Our study had some limitations. Tracking the levels of cobalt, acetylcholine, and thrombin in the brain might have added insights to our findings; however, the assessed parameters were sufficient to serve our aim, without complicating the interpretation of results.

## Conclusions

CoCl_2_ replicated the motor dysfunction, neurodegeneration of SN and STR, and defective dopaminergic signaling associated with other parkinsonian models. Our CIP model illustrated the crosstalk between the dopaminergic, adenosinergic, thrombin-related, and oxidative stress pathways. The CIP model might not exemplify the primary implication of dopaminergic deficit in the triggered motor dysfunction but rather fits a model in which defective dopaminergic neurotransmission is secondary to the non-dopaminergic effect.

BM+DE improved motor dysfunction, with partial recovery of neuronal insults in SN and STR, by resolving the defective DA and D2, increasing A1/A2A, and restoring PAR1, ET1, and MDA/GSH. Employing BM+DE in other models of parkinsonian is warranted, especially when thromboembolic events and oxidative stress are anticipated.

## References

[1] Riley BE, Gardai SJ, Emig-Agius D (2014). Systems-based analyses of brain regions functionally impacted in Parkinson's disease reveals underlying causal mechanisms. PLoS One.

[2] Rizzi G, Tan KR (2017). Dopamine and acetylcholine, a circuit point of view in Parkinson’s disease. Front Neural Circuits.

[3] (2024). NICE guideline: Parkinson's disease in adults.. http://www.nice.org.uk/guidance/ng71.

[4] Ferguson LW, Rajput AH, Rajput A (2016). Early-onset vs. late-onset Parkinson’s disease: a clinical-pathological study. Can J Neurol Sci.

[5] Song Z, Liu S, Li X, Zhang M, Wang X, Shi Z, Ji Y (2022). Prevalence of Parkinson’s disease in adults aged 65 years and older in China: a multicenter population-based survey. Neuroepidemiology.

[6] Phani S, Loike JD, and Perzedborski S (2012). Neurodegeneration and inflammation in Parkinson's disease. Parkinsonism Relat Disord.

[7] Olanow CW, Koller WC (1998). An algorithm (decision tree) for the management of Parkinson's disease: treatment guidelines. Neurology.

[8] Brocks DR (1999). Anticholinergic drugs used in Parkinson's disease: an overlooked class of drugs from a pharmacokinetic perspective. J Pharm Pharm Sci.

[9] Yaar R, Jones MR, Chen JF, Ravid K (2005). Animal models for the study of adenosine receptor function. J Cell Physiol.

[10] Prinster SC, Hague C, Hall RA (2005). Heterodimerization of g protein-coupled receptors: specificity and functional significance. Pharmacol Rev.

[11] Fuxe K, Agnati LF, Jacobsen K (2003). Receptor heteromerization in adenosine A2A receptor signaling: relevance for striatal function and Parkinson's disease. Neurology.

[12] Preston Z, Lee K, Widdowson L, Freeman TC, Dixon AK, Richardson PJ (2000). Adenosine receptor expression and function in rat striatal cholinergic interneurons. Br J Pharmacol.

[13] Lanznaster D, Massari CM, Marková V (2019). Adenosine A(1)-A(2a) receptor-receptor interaction: contribution to guanosine-mediated effects. Cells.

[14] Bennett KA, Tehan B, Lebon G, Tate CG, Weir M, Marshall FH, Langmead CJ (2013). Pharmacology and structure of isolated conformations of the adenosine A₂A receptor define ligand efficacy. Mol Pharmacol.

[15] Sanyal J, Bandyopadhyay SK, Banerjee TK, Mukherjee SC, Chakraborty DP, Ray BC, Rao VR (2009). Plasma levels of lipid peroxides in patients with Parkinson's disease. Eur Rev Med Pharmacol Sci.

[16] Liddell JR, White AR (2018). Nexus between mitochondrial function, iron, copper and glutathione in Parkinson's disease. Neurochem Int.

[17] Mischley LK, Standish LJ, Weiss NS, Padowski JM, Kavanagh TJ, White CC, Rosenfeld ME (2016). Glutathione as a biomarker in Parkinson’s disease: associations with aging and disease severity. Oxid Med Cell Longev.

[18] Chen YF, Oparil S (2000). Endothelin and pulmonary hypertension. J Cardiovasc Pharmacol.

[19] Yan Y, Fu J (2021). Plasma ApoA-1 and endothelin-1 levels changes in early Parkinson disease and its relationship with cognitive function and cerebral white matter structure change. Pak J Pharm Sci.

[20] Ebrahimi S, Jaberi N, Avan A, Ryzhikov M, Keramati MR, Parizadeh MR, Hassanian SM (2017). Role of thrombin in the pathogenesis of central nervous system inflammatory diseases. J Cell Physiol.

[21] Ishida Y, Nagai A, Kobayashi S, Kim SU (2006). Upregulation of protease-activated receptor-1 in astrocytes in Parkinson disease: astrocyte-mediated neuroprotection through increased levels of glutathione peroxidase. J Neuropathol Exp Neurol.

[22] Reuland CJ, Church FC (2020). Synergy between plasminogen activator inhibitor-1, α-synuclein, and neuroinflammation in Parkinson's disease. Med Hypotheses.

[23] Rizzetti MC, Liberini P, Zarattini G (2009). Loss of sight and sound. Could it be the hip?. Lancet.

[24] Santner A, Uversky VN (2011). α-synuclein and metals. Protein Folding and Metal Ions: Mechanisms, Biology and Disease.

[25] Hartmann A, Hannemann F, Lützner J, Seidler A, Drexler H, Günther KP, Schmitt J (2013). Metal ion concentrations in body fluids after implantation of hip replacements with metal-on-metal bearing--systematic review of clinical and epidemiological studies. PLoS One.

[26] Guan D, Su Y, Li Y, Wu C, Meng Y, Peng X, Cui Y (2015). Tetramethylpyrazine inhibits CoCl2 -induced neurotoxicity through enhancement of Nrf2/GCLc/GSH and suppression of HIF1α/NOX2/ROS pathways. J Neurochem.

[27] Lan AP, Chen J, Chai ZF, Hu Y (2016). The neurotoxicity of iron, copper and cobalt in Parkinson's disease through ROS-mediated mechanisms. Biometals.

[28] Zhong X, Lin R, Li Z, Mao J, Chen L (2014). Effects of Salidroside on cobalt chloride-induced hypoxia damage and mTOR signaling repression in PC12 cells. Biol Pharm Bull.

[29] Ullah N, Qureshi MT, Toufiq AM (2021). Effect of cobalt doping on the structural, optical and antibacterial properties of α-MnO 2 nanorods. Appl Phys A.

[30] Kotake-Nara E, Saida K (2006). Endothelin-2/vasoactive intestinal contractor: regulation of expression via reactive oxygen species induced by CoCl2, and Biological activities including neurite outgrowth in PC12 cells. Sci World J.

[31] Mou YH, Yang JY, Cui N, Wang JM, Hou Y, Song S, Wu CF (2012). Effects of cobalt chloride on nitric oxide and cytokines/chemokines production in microglia. Int Immunopharmacol.

[32] Carey RJ, De Veaugh-Geiss J (1982). Chronic benztropine and haloperidol administration induce behaviorally equivalent pharmacological hypersensitivities separately but not in combination. Psychopharmacology (Berl).

[33] Feldman S, Putcha L (1977). Effect of anti-parkinsonism drugs on gastric emptying and intestinal transit in the rat. Pharmacology.

[34] Ware KM, Vance JC, Muni N (2015). Oral warfarin and the thrombin inhibitor dabigatran increase blood pressure in rats: hidden danger of anticoagulants?. Am J Hypertens.

[35] Shrivastava K, Bansal A, Singh B, Sairam M, Ilavazhagan G (2010). Sub-chronic oral toxicity study in Sprague-Dawley rats with hypoxia mimetic cobalt chloride towards the development of promising neutraceutical for oxygen deprivation. Exp Toxicol Pathol.

[36] Fleming SM, Zhu C, Fernagut PO, Mehta A, DiCarlo CD, Seaman RL, Chesselet MF (2004). Behavioral and immunohistochemical effects of chronic intravenous and subcutaneous infusions of varying doses of rotenone. Exp Neurol.

[37] Sereniki A, Linard C, Silva SN (2016). Schinus terebinthifolius administration prevented behavioral and biochemical alterations in a rotenone model of Parkinson's disease. Rev bras farmacogn.

[38] Woodlee MT, Kane JR, Chang J, Cormack LK, Schallert T (2008). Enhanced function in the good forelimb of hemi-parkinson rats: compensatory adaptation for contralateral postural instability?. Exp Neurol.

[39] Allred RP, Adkins DL, Woodlee MT (2008). The vermicelli handling test: A simple quantitative measure of dexterous forepaw function in rats. J Neurosci Methods.

[40] Khaing ZZ, Geissler SA, Schallert T, Schmidt CE (2013). Assessing forelimb function after unilateral cervical SCI using novel tasks: Limb step-alternation, postural instability and pasta handling. J Vis Exp.

[41] Tieu K (2011). A guide to neurotoxic animal models of Parkinson's disease. Cold Spring Harb Perspect Med.

[42] Adams B, Nunes JM, Page MJ (2019). Parkinson’s disease: A systemic inflammatory disease accompanied by bacterial Inflammagens. Front Aging Neurosci.

[43] Kerimoglu S, Onay A, Guvercin Y, Çitlak A, Yenilmez E, Kerimoglu G (2015). The effects of dabigatran etexilate on fracture healing in rats: An experimental study. Indian J Orthop.

[44] Scridon A, Perian M, MĂrginean A (2018). Plasma lipids affect dabigatran etexilate anticoagulation in rats with unbalanced diabetes mellitus. J Diabetes.

[45] Avci S, Gungor H, Kumru AS (2021). Effects of apixaban, rivaroxaban, dabigatran and enoxaparin on histopathology and laboratory parameters in Achilles tendon injury: An in vivo study. Saudi J Med Med Sci.

[46] Shikamoto Y, Morita T (1999). Expression of factor X in both the rat brain and cells of the central nervous system. FEBS letters.

[47] Palakurthi B, Burugupally SP (2019). Postural instability in Parkinson’s disease: A review. Brain Sci.

[48] Akinrinde AS, Adebiyi OE (2019). Neuroprotection by luteolin and gallic acid against cobalt chloride-induced behavioural, morphological and neurochemical alterations in Wistar rats. Neurotoxicology.

[49] Cannon JR, Tapias V, Na HM, Honick AS, Drolet RE, Greenamyre JT (2009). A highly reproducible rotenone model of Parkinson's disease. Neurobiol Dis.

[50] Fasano A, Mazzoni A, Falotico E (2022). Reaching and grasping movements in Parkinson’s disease: A review. J Parkinsons Dis.

[51] Berg D, Adler CH, Bloem BR (2018). Movement disorder society criteria for clinically established early Parkinson's disease. Mov Disord.

[52] Kandil EA, Sayed RH, Ahmed LA, Abd El Fattah MA, El-Sayeh BM (2018). Modulatory role of Nurr1 activation and thrombin inhibition in the neuroprotective effects of dabigatran etexilate in rotenone-induced Parkinson’s disease in rats. Mol Neurobiol.

[53] Kane JR, Ciucci MR, Jacobs AN (2011). Assessing the role of dopamine in limb and cranial-oromotor control in a rat model of Parkinson's disease. J Commun Disord.

[54] Apostoli P, Catalani S, Zaghini A (2013). High doses of cobalt induce optic and auditory neuropathy. Exp Toxicol Pathol.

[55] Gómez-Arnaiz S, Tate RJ, Grant MH (2022). Cobalt neurotoxicity: Transcriptional effect of elevated cobalt blood levels in the rodent brain. Toxics.

[56] Fulton S, Thibault D, Mendez JA (2011). Contribution of Kv1.2 voltage-gated potassium channel to D2 autoreceptor regulation of axonal dopamine overflow. J Biol Chem.

[57] McFarland NR, Hess CW (2017). Recognizing Atypical Parkinsonisms: "Red Flags" and Therapeutic Approaches. Semin Neurol.

[58] Varani K, Vincenzi F, Tosi A (2010). A2A adenosine receptor overexpression and functionality, as well as TNF-alpha levels, correlate with motor symptoms in Parkinson's disease. FASEB J.

[59] Ferré S, Quiroz C, Rea W, Guitart X, García-Borreguero D (2019). Adenosine mechanisms and hypersensitive corticostriatal terminals in restless legs syndrome. Rationale for the use of inhibitors of adenosine transport. Adv Pharmacol.

[60] Ferré S, O'Connor WT, Snaprud P (1994). Antagonistic interaction between adenosine A2A receptors and dopamine D2 receptors in the ventral striopallidal system. Implications for the treatment of schizophrenia. Neuroscience.

[61] Ismayilova N, Crossman A, Verkhratsky A, Brotchie J (2004). Effects of adenosine A1, dopamine D1 and metabotropic glutamate 5 receptors-modulating agents on locomotion of the reserpinised rats. Eur J Pharmacol.

[62] Borycz J, Pereira MF, Melani A (2007). Differential glutamate-dependent and glutamate-independent adenosine A1 receptor-mediated modulation of dopamine release in different striatal compartments. J Neurochem.

[63] Liu A, Wijesurendra RS, Francis JM, Robson MD, Neubauer S, Piechnik SK, Ferreira VM (2016). Adenosine stress and rest T1 mapping can differentiate between ischemic, infarcted, remote, and normal myocardium without the need for gadolinium contrast agents. JACC Cardiovasc Imaging.

[64] Augusto E, Matos M, Sévigny J (2013). Ecto-5'-nucleotidase (CD73)-mediated formation of adenosine is critical for the striatal adenosine A2A receptor functions. J Neurosci.

[65] Sarasola LI, Del Torrent CL, Pérez-Arévalo A (2022). The ADORA1 mutation linked to early-onset Parkinson's disease alters adenosine A(1)-A(2A) receptor heteromer formation and function. Biomed Pharmacother.

[66] Stockwell J, Chen Z, Niazi M, Nosib S, Cayabyab FS (2016). Protein phosphatase role in adenosine A1 receptor-induced AMPA receptor trafficking and rat hippocampal neuronal damage in hypoxia/reperfusion injury. Neuropharmacology.

[67] Choi SH, Lee DY, Ryu JK, Kim J, Joe EH, Jin BK (2003). Thrombin induces nigral dopaminergic neurodegeneration in vivo by altering expression of death-related proteins. Neurobiol Dis.

[68] Chen R, Cao X, Luo W, Yang H, Luo X, Yu J, Luo J (2020). Dabigatran suppresses par-1/SphK/S1P activation of astrocytes in experimental autoimmune encephalomyelitis model. Front Mol Neurosci.

[69] Wu CC, Wang WY, Wei CK, Teng CM (2011). Combined blockade of thrombin anion binding exosite-1 and PAR4 produces synergistic antiplatelet effect in human platelets. Thromb Haemost.

[70] Cannon JR, Keep RF, Schallert T, Hua Y, Richardson RJ, Xi G (2006). Protease-activated receptor-1 mediates protection elicited by thrombin preconditioning in a rat 6-hydroxydopamine model of Parkinson's disease. Brain Res.

[71] Sveshnikova AN, Balatskiy AV, Demianova AS (2016). Systems biology insights into the meaning of the platelet's dual-receptor thrombin signaling. J Thromb Haemost.

[72] Jain A, Olovsson M, Burton GJ, Yung HW (2012). Endothelin-1 induces endoplasmic reticulum stress by activating the PLC-IP(3) pathway: implications for placental pathophysiology in preeclampsia. Am J Pathol.

[73] Ferrari CC, Tarelli R (2011). Parkinson's disease and systemic inflammation. Parkinsons Dis.

[74] Chiang PL, Chen HL, Lu CH (2017). White matter damage and systemic inflammation in Parkinson's disease. BMC Neurosci.

[75] Michinaga S, Koyama Y (2017). Protection of the blood-brain barrier as a therapeutic strategy for brain damage. Biol Pharm Bull.

[76] Zheng F, Luo Z, Zheng C (2019). Comparison of the neurotoxicity associated with cobalt nanoparticles and cobalt chloride in Wistar rats. Toxicol Appl Pharmacol.

[77] Hauser DN, Hastings TG (2013). Mitochondrial dysfunction and oxidative stress in Parkinson's disease and monogenic parkinsonism. Neurobiol Dis.

[78] Ahlskog JE, Uitti RJ, Low PA, Tyce GM, Nickander KK, Petersen RC, Kokmen E (1995). No evidence for systemic oxidant stress in Parkinson's or Alzheimer's disease. Mov Disord.

[79] Çokal BG, Yurtdaş M, Güler SK (2017). Serum glutathione peroxidase, xanthine oxidase, and superoxide dismutase activities and malondialdehyde levels in patients with Parkinson's disease. Neurol Sci.

[80] Anandhan A, Tamilselvam K, Vijayraja D, Ashokkumar N, Rajasankar S, Manivasagam T (2010). Resveratrol attenuates oxidative stress and improves behaviour in 1 -methyl-4-phenyl-1,2,3,6-tetrahydropyridine (MPTP) challenged mice. Ann Neurosci.

[81] Kikuoka R, Miyazaki I, Kubota N (2020). Mirtazapine exerts astrocyte-mediated dopaminergic neuroprotection. Sci Rep.

[82] Latif S, Jahangeer M, Maknoon Razia D (2021). Dopamine in Parkinson's disease. Clin Chim Acta.

[83] Bahar I, Elay G, Başkol G, Sungur M, Donmez-Altuntas H (2018). Increased DNA damage and increased apoptosis and necrosis in patients with severe sepsis and septic shock. J Crit Care.

[84] Hwang O (2013). Role of oxidative stress in Parkinson's disease. Exp Neurobiol.

[85] Sohrabi T, Mirzaei-Behbahani B, Zadali R, Pirhaghi M, Morozova-Roche LA, Meratan AA (2023). Common mechanisms underlying α-synuclein-induced mitochondrial dysfunction in Parkinson’s disease. J Mol Biol.

